# Transgene-free direct conversion of murine fibroblasts into functional muscle stem cells

**DOI:** 10.1038/s41536-023-00317-z

**Published:** 2023-08-08

**Authors:** Xhem Qabrati, Inseon Kim, Adhideb Ghosh, Nicola Bundschuh, Falko Noé, Andrew S. Palmer, Ori Bar-Nur

**Affiliations:** 1 Laboratory of Regenerative and Movement Biology, Department of Health Sciences and Technology, ETH Zurich, Schwerzenbach, Switzerland; 2 Functional Genomics Center Zurich, ETH Zurich and University of Zurich, Zurich, Switzerland; 3 Present Address: Institute for Health and Sport, Victoria University, Footscray, VIC Australia

**Keywords:** Reprogramming, Regeneration, Muscle stem cells

## Abstract

Transcription factor-based cellular reprogramming provides an attractive approach to produce desired cell types for regenerative medicine purposes. Such cellular conversions are widely dependent on viral vectors to efficiently deliver and express defined factors in target cells. However, use of viral vectors is associated with unfavorable genomic integrations that can trigger deleterious molecular consequences, rendering this method a potential impediment to clinical applications. Here, we report on a highly efficient transgene-free approach to directly convert mouse fibroblasts into induced myogenic progenitor cells (iMPCs) by overexpression of synthetic MyoD-mRNA in concert with an enhanced small molecule cocktail. First, we performed a candidate compound screen and identified two molecules that enhance fibroblast reprogramming into iMPCs by suppression of the JNK and JAK/STAT pathways. Simultaneously, we developed an optimal transfection protocol to transiently overexpress synthetic MyoD-mRNA in fibroblasts. Combining these two techniques enabled robust and rapid reprogramming of fibroblasts into Pax7 positive iMPCs in as little as 10 days. Nascent transgene-free iMPCs proliferated extensively in vitro, expressed a suite of myogenic stem cell markers, and could differentiate into highly multinucleated and contractile myotubes. Furthermore, using global and single-cell transcriptome assays, we delineated gene expression changes associated with JNK and JAK/STAT pathway inhibition during reprogramming, and identified in iMPCs a *Pax7*^+^ stem cell subpopulation resembling satellite cells. Last, transgene-free iMPCs robustly engrafted skeletal muscles of a Duchenne muscular dystrophy mouse model, restoring dystrophin expression in hundreds of myofibers. In summary, this study reports on an improved and clinically safer approach to convert fibroblasts into myogenic stem cells that can efficiently contribute to muscle regeneration in vivo.

## Introduction

Reprogramming cell fate via transcription factor overexpression has been a seminal milestone in cell biology^1^. To achieve lineage conversion, efficient delivery of cell-type-specific genes into target cells has been pivotal for the success of reprogramming trials^1^. For example, the conversion of fibroblasts into skeletal muscle cells by overexpression of the myogenic determination 1 (MyoD) gene was dependent on an efficient retroviral delivery system that was the pinnacle of viral vector technology at that time^2^. Similarly, retroviruses have been employed to overexpress in fibroblasts dozens of transcription factors in tandem, thereby unfolding a gene combination that induces pluripotency in somatic cells^3^. While retroviruses were central to the success of these breakthrough achievements, retroviral particles transduce only dividing cells, an attribute which presents an impediment towards transduction of certain cell types, rendering lentiviral vectors that transduce both dividing and non-dividing cells an improved method^4,5^. However, transducing vectors can integrate near or into coding regions of the genome, potentially eliciting unfavorable expression or suppression of endogenous genes, including activation of oncogenes^6–8^. Moreover, viral transduction can evoke an immune response, high toxicity, extensive molecular damage or recombination events that can produce replication-competent viruses^4,9^. These unfavorable attributes present challenges when utilizing viral vectors for gene therapy trials, or reprogrammed cells for cell-based therapies^10,11^. As a result, extensive effort has been taken in recent years to develop DNA integration-free delivery methods^10,11^. In respect to reprogramming cell fate, these efforts have included episomal DNA vectors, Adeno and Sendai viruses, CRISPR-based gene activation and protein delivery^12–19^. Most notably, since the fundamental discovery that incorporation of modified nucleosides into synthetic mRNA mitigates cellular immune reactions against exogenous mRNA molecules^20,21^, considerable research has been directed towards employing mRNA-based technology to overexpress transcription factors of interest for induction of cellular conversion^11^. These efforts demonstrated the feasibility of using mRNA-based technology to alter cell fate, however, required optimization to efficiently overexpress genes of interest and prevent cell death that is associated with use of mRNA molecules^22^. Nonetheless, several studies have reported mRNA-based conversion of fibroblasts into induced pluripotent stem cells (iPSCs) as well as other cell types^23–29^. Recent studies have further utilized mRNA-based technology to directly differentiate PSCs into a variety of lineages^24,30–33^.

An additional approach to enhance or govern cell fate conversion is the administration of small molecules that target key signaling pathways^34^. For example, small molecule treatment has been widely used in conjunction with transcription factor overexpression to enhance cellular conversion into multiple cell types including iPSCs, neurons, hepatocytes, and cardiomyocytes^34^. Moreover, small molecules alone can induce cellular conversion, negating the use of transcription factors and associated viral vectors^34^. Prominent examples from recent years include reprogramming of fibroblasts into cardiac, neural and pluripotent cells solely using compound treatment^35–39^.

We and others have previously established a method to reprogram mouse fibroblasts into induced myogenic progenitor cells (iMPCs) that resemble satellite cells, the stem cells of skeletal muscle tissue^40–42^. To this end, MyoD is overexpressed in fibroblasts using a doxycycline (dox)-inducible lentiviral system in conjunction with three small molecules, the cyclic AMP agonist Forskolin (F), the TGF-β receptor inhibitor RepSox (R) and the GSK3 inhibitor CHIR99210 (C) (abbreviated as “F/R/C”)^40^. In contrast to the iMPC reprogramming system, conventional transdifferentiation solely by MyoD has been shown to give rise to non-proliferative postmitotic muscle cells^2,40^. To elicit myogenic transdifferentiation, MyoD functions as a pioneer transcription factor that binds E-Box DNA motif elements, inducing chromatin rewiring and activating a large number of skeletal muscle-associated genes^43–48^. Notably and unlike transdifferentiated myotubes, directly reprogrammed iMPCs form a heterogeneous myogenic culture consisting of skeletal muscle stem, progenitor and differentiated cells, that are passaged in tandem and can efficiently engraft wild type (WT) and dystrophic muscles of Duchenne muscular dystrophy (DMD) mice in vivo^40^. However, a potential caveat for utilizing iMPCs for regenerative medicine purposes involves the use of lentiviral particles to overexpress MyoD in target cells^40–42^. Additionally, the prominent heterogeneity of iMPC cultures may present a potential obstacle for cell-based therapy and could benefit from means to cultivate the stem cell subset of iMPCs more homogenously.

To address these limitations, here we aimed to identify additional compounds that can preferentially increase the stem cell population of iMPCs during conventional reprogramming via lentiviral MyoD overexpression in concert with F/R/C treatment. In addition, we aimed to develop and optimize a synthetic MyoD-mRNA delivery system that in conjunction with F/R/C supplementation can generate integration-free iMPCs. By combining these two approaches, we then set out to trial production of transgene-free iMPCs that contain a higher number of myogenic stem cells, can proliferate extensively in vitro and efficiently form multinucleated myotubes. Last, we investigated whether transplantation of transgene-free iMPCs can contribute to in vivo dystrophin restoration in skeletal muscles of DMD mice.

## Results

### Inhibition of the JNK and JAK/STAT pathways enhances fibroblast conversion to iMPCs

**Fig. 1:**
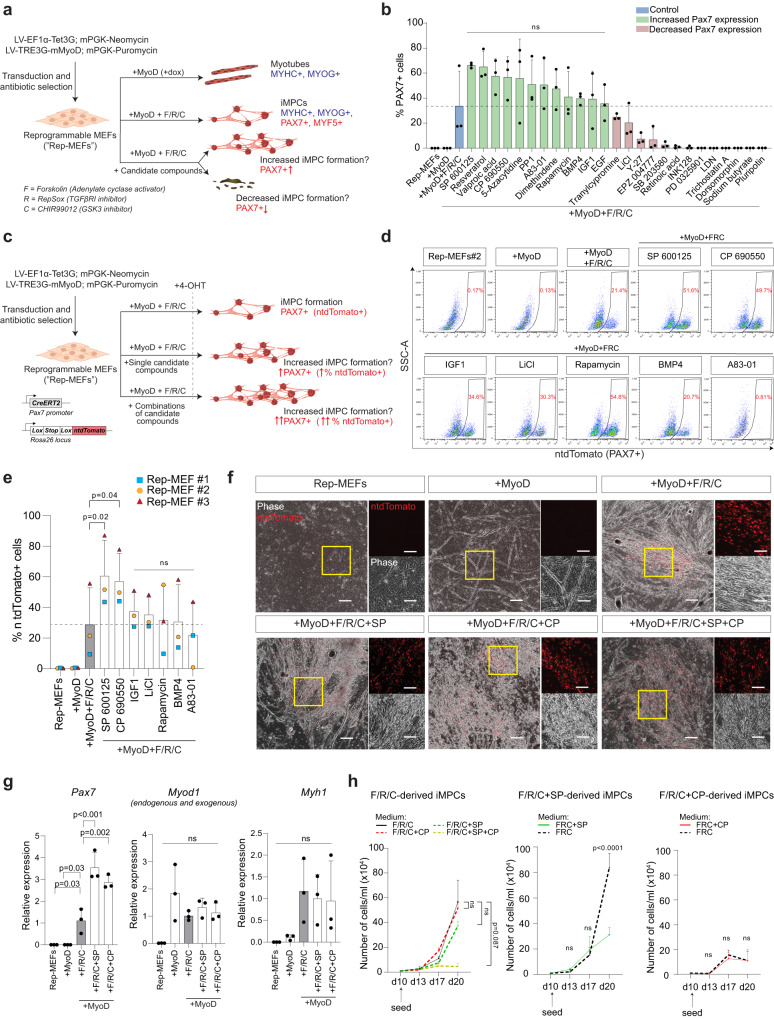
JNK and JAK/STAT inhibition enhance fibroblast conversion into iMPCs. **a** A schematic illustrating compound screening strategy to uncover facilitators of iMPC reprogramming. LV, lentiviruses; iMPCs, induced myogenic progenitor cells; MEFs, mouse embryonic fibroblasts. **b** Quantification of small molecule screen at day 14 of reprogramming as assessed by immunofluorescence of PAX7^+^ cells. *N* = 3, each dot represents one field of view of an assessed cell line. Data are shown as mean ± SD. Significance was assessed relative to the “+MyoD+F/R/C condition” (ns=non-significant) by unpaired two-tailed *t* tests. **c** Schematic depicting experimental design. **d** FACS plots showing percentages of ntdTomato^+^/PAX7^+^ cells on day 10 of reprogramming of *Pax7-CreERT2; R26-LSL-ntdTomato* MEFs using the outlined conditions. Cells were labeled with 4-OHT two days prior to analysis. **e** A graph showing FACS-quantification of ntdTomato^+^/PAX7^+^ cells on day 10 of reprogramming of *Pax7-CreERT2; R26-LSL-ntdTomato* MEFs using the indicated cell lines and conditions. Cells were labeled with 4-OHT two days prior to analysis. *N* = 3, each symbol represents a different cell line. Data are shown as mean ± SD. Significance was determined using a linear model employing group-wise comparisons between treatment groups and percentage of ntdTomato^+^ cells (excluding “Rep-MEFs” and “+MyoD” conditions). One-sided *p*-values are shown after adjustment for family-wise error rate using the Bonferroni–Holm method. **f** Representative images showing MEFs subjected to the indicated conditions on day 10 of reprogramming. Scale bar, 200 μm; scale bar inlay, 100 μm. **g** RT-qPCR analysis of Rep-MEFs #1-3 subjected to the indicated conditions at day 10 of reprogramming. Expression is shown relative to the “+MyoD+F/R/C” condition. *N* = 3, each dot represents a different cell line. Data are shown as mean ± SD. Significance is determined by ordinary one-way ANOVA using Dunnett’s multiple comparisons test taking “MyoD+F/R/C” as a control condition, ns=non-significant. **h** Growth curves of iMPCs at passage 5 (P5) derived by the indicated conditions and exposed to medium containing the outlined small molecules for 10–20 days. *N* = 3 independent experiments. Significance is determined by two-way ANOVA using Tukey’s multiple comparisons test with a single pooled variance. Data are shown as mean ± SD.

We commenced our investigation by aiming to identify an optimized small molecule cocktail that can augment iMPC production from fibroblasts (Fig. 1a). We hypothesized that manipulation of additional signaling pathways, in concert with the conventional F/R/C treatment, may increase the conversion efficiency of fibroblasts into iMPCs (Fig. 1a). To investigate this possibility, we opted to trial addition of candidate small molecules to the previously reported MyoD+F/R/C protocol^40^, employing immunofluorescence for Pax7 expression as a readout for successful reprogramming, as this transcription factor is highly expressed in muscle stem cells^49^. We assembled a candidate compound library based on a literature search consisting of 25 small molecules, most of which have been previously shown to play important roles in the modulation of signaling pathways in a variety of lineage reprogramming studies^34^. For the first round of compound screening, reprogrammable mouse embryonic fibroblasts (Rep-MEFs) containing a dox-dependent MyoD overexpression cassette were subjected to MyoD, MyoD+F/R/C and MyoD+F/R/C+candidate compounds for 14 days^40^, at which time point the cultures were analyzed for PAX7 expression (Figs. 1a and S1a). Surprisingly, the addition of nine small molecules impeded the formation of PAX7^+^ cells, suggesting the involvement of their respective molecular targets in iMPC formation (Figs. 1b and S1a). Furthermore, several compounds did not affect the number of PAX7^+^ cells, whereas a few compounds enhanced iMPC formation as judged by an increase in the number of PAX7^+^ cells (Fig. 1b). The latter included the c-Jun N-terminal kinase (JNK) inhibitor SP600125 (SP), the Janus kinase (JAK) inhibitor CP690550 (CP), the SIRT1 activator Resveratrol and the epigenetic modulators valproic acid and 5-azacytidine (Fig. 1b).

The first round of small molecule screening pointed towards promising candidates for further investigation. As iMPCs consist of three-dimensional clusters, rendering an unbiased quantification by immunofluorescence challenging, we sought an additional method to assess for reprogramming efficiency. We opted to employ Rep-MEFs carrying *Pax7-CreERT2; Rosa26-Lox-STOP-Lox-ntdTomato (R26-LSL-ntdTomato)* alleles that prospectively label *Pax7* expressing cells and their progeny upon 4-hydroxytamoxifen (4-OHT) administration (Fig. 1c)^40,50^. We selected several compounds for further testing based on the initial screen or their involvement in muscle regeneration including SP, CP, Rapamycin, IGF-1, LiCl, BMP4, and A83-01. A Fluorescence Activated Cell Sorting (FACS)-analysis at day 10 of the reprogramming course revealed that the most substantial increase in ntdTOMATO^+^/PAX7^+^ cells was under the SP and CP conditions, demonstrating on average around 55–60% PAX7^+^ cells in comparison to around 30% PAX7^+^ cells under the conventional MyoD+F/R/C condition (Figs. 1d, e and S1b for gating strategy). Overall, using three different Rep-MEF lines, a consistent upregulation of PAX7^+^ cells under SP and CP treatment was observed, albeit with a high variation between MEF lines (Fig. 1e). As such, we wished to confirm our observation in respect to PAX7 upregulation by utilizing a *Pax7-nuclear (n) GFP* genetic reporter during reprogramming^51^. We repeated the initial screen using the 25 compounds and similarly documented a higher number of PAX7^+^ cells under the SP or CP treatment at day 14 (Fig. S1d, e, c for gating strategy). Together, these analyses pointed towards improved iMPC formation as judged by PAX7 upregulation with either SP or CP treatment in concert with MyoD+F/R/C. As such, we decided to focus our efforts on characterizing the reprogramming process in the presence of these two inhibitors for the remainder of this study.

As the next step, we determined that individual or combined addition of the two inhibitors with MyoD + F/R/C does not have a substantial effect on the appearance of iMPC clones during reprogramming (Fig. 1f). Interestingly, the addition of either SP or CP increased *Pax7* mRNA transcript level by Real Time quantitative PCR (RT-qPCR), however did not alter the expression of the myogenic differentiation gene *Myh1* (Fig. 1g). Accordingly, a Western blot analysis documented a higher PAX7 protein expression under MyoD+F/R/C + SP, CP or SP + CP in comparison to MyoD + F/R/C (Fig. S2a). We also noted a slight increase in the number of PAX7^+^ cells upon SP and CP treatment at earlier reprogramming time points, prompting us to analyze iMPC formation at day 10 during several subsequent experiments in this study (Fig. S2b). We then assessed the fusion index at day 10 of reprogramming and noted that it was similar for all conditions aside from a lower propensity under MyoD overexpression alone (Fig. S2c, d). Moreover, we assessed PAX7 upregulation using additional *Pax7-CreERT2; R26-LSL-ntdTomato* Rep-MEF lines with all possible combinations of F, R, and C together with either SP, CP or SP + CP. We observed that the inhibitors increased the number of PAX7^+^ cells solely with F/R/C but not other small molecule combinations (Fig. S3a–c). An RT-qPCR analysis with additional Rep-MEF lines confirmed the *Pax7* upregulation upon SP or SP + CP treatment, however, the expression of differentiation myogenic genes was about the same, aside from lower *Myf6* expression (Fig. S3d).

To investigate whether SP or CP treatment increases cell proliferation rate, we performed an EdU analysis on day 10 of reprogramming and observed a slightly elevated number of EdU^+^ cells when SP or CP were administered alone or in tandem (Fig. S4a). To assess whether this observation might be due to proliferating PAX7^+^ cells, we performed an immunostaining for PAX7 and the proliferation marker MKI67, documenting an increase in proliferating PAX7^+^ cells under SP or SP + CP treatment at day 10 (Fig. S4b, c). Last, we wished to assess the growth rate of established iMPC clones generated with F/R/C, SP or CP by a growth curve analysis. To this end, we first cultured F/R/C-derived clones under individual or combined presence of the two inhibitors and documented reduced growth rate when SP and CP were added together to the conventional F/R/C medium (Fig. 1h). Furthermore, continuous culture of SP-derived iMPCs in F/R/C + SP conditions showed reduced growth rate in comparison to the conventional F/R/C condition, whereas the growth rate of CP treated iMPCs remained unchanged (Fig. 1h). Notably, continuous passaging in the presence of SP, CP or SP + CP oftentimes reduced the growth of iMPCs and altered their morphology, precipitating less or more myotubes, however removal of these compounds from the medium could restore morphological attributes (Fig. S4d). Altogether, we report the identification of two inhibitors that enhance PAX7 expression and iMPC formation from fibroblasts by suppression of the JNK and JAK/STAT signaling pathways. Under certain conditions, extended culture of iMPCs in the presence of these small molecules was impaired, suggesting their favorable role in enhancing iMPC derivation, however not long-term propagation.

### Transgene-free iMPCs produced by synthetic MyoD-mRNA and small molecules

Lentivirus-driven MyoD overexpression is critical for iMPC formation, as F/R/C treatment alone fails to produce iMPCs from fibroblasts^40,41^. However, the use of lentiviruses for iMPC production carries associated risks in the form of insertional mutagenesis, poor control over copy number and a risk for the generation of replication-competent viruses. As such, we reasoned that delivery of synthetic MyoD-mRNA could pose a more suitable and safer alternative to lentiviral vectors by enabling an integration-free and transient overexpression of MyoD in fibroblasts.

**Fig. 2:**
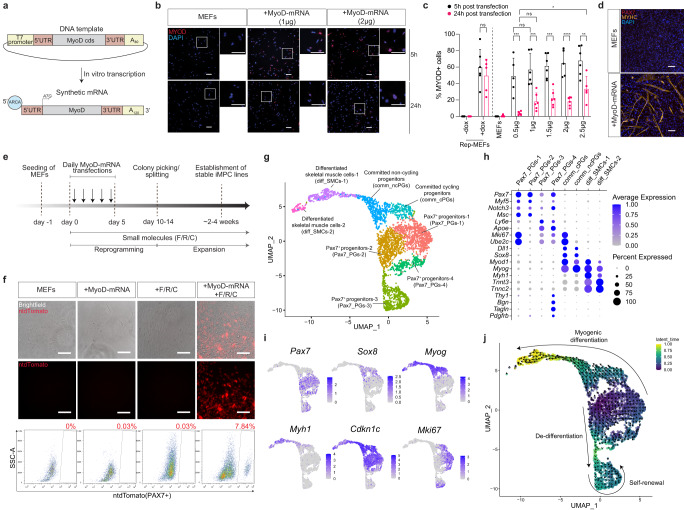
Reprogramming fibroblasts into iMPCs with synthetic MyoD-mRNA. **a** A schematic depicting the plasmid construct utilized to generate synthetic MyoD-mRNA. Cds, coding sequence; UTR, untranslated region; ARCA, anti-reverse cap analog. **b** Representative immunofluorescence images of MYOD expression in MEFs transfected with MyoD-mRNA either 5 or 24 h post treatment. Scale bar, 100 μm. Scale bar inlay, 100 μm. **c** Quantification of MYOD^+^ cells as assessed by immunofluorescence. Data are shown as mean ± SD. *N* = 3, either 1 or 2 random fields of view were quantified for each cell line. Significance is determined by a mixed-effects analysis using Tukey’s multiple comparison’s test with a single pooled variance, ns=non-significant; *p < 0.05, **p < 0.01, ***p < 0.001, ****p < 0.0001. **d** Immunofluorescence staining for the indicated proteins in MEFs transfected 4x with synthetic MyoD-mRNA and stained at day 5. Scale bar, 100 μm. **e** Timeline of experimental design to produce transgene-free iMPCs. **f** Top: Images showing ntdTomato^+^ cells following reprogramming of *Pax7-CreERT2; R26-LSL-ntdTomato* MEFs with MyoD-mRNA+F/R/C at day 15. Cells were labeled with 4-OHT three days prior to analysis. Bottom: FACS-analysis of ntdTomato^+^ cells corresponding to the experiment shown above. Scale bar, 100 μm. **g** A UMAP projection showing 3517 cells comprising a MyoD-mRNA-derived iMPC clone at passage 5. **h** Dot plot indicating gene expression level of selected genes in each respective cluster. Average gene expression across all cells in each cluster is shown using a color scale. The percentage of cells expressing the indicated genes is shown as dot size. **i** A UMAP projection showing the expression level of selected myogenic genes across all cells via a color gradient. **j** A UMAP projection of single-cell trajectory as indicated by RNA velocity and colored by the latent time of the underlying cellular process.

To explore this possibility and test the potential of synthetic MyoD-mRNA to produce transgene-free iMPCs, we designed a plasmid for in vitro mRNA transcription. This plasmid harbored the murine MyoD coding sequence flanked at the 5′ end by a T7 promoter before a 5′ UTR, and at the 3′ end by the 3′ UTR of the *alpha globin* gene in conjunction with a poly-A tail^24^ (Fig. 2a). In the nascent in vitro transcribed mRNA, we incorporated a 5′ anti-reverse cap analog (ARCA) and a 120 bp poly-A tail to enable increased mRNA stability^20,21,52^ (Fig. 2a). Furthermore, we substituted the nucleosides uridine with pseudouridine-5′-triphosphate and cytidine with 5-methylcytidine-5′-triphosphate, respectively^20,21^. Collectively, these modifications are widely known to be important for mitigating inflammatory responses due to activation of the innate immune system following mRNA delivery, in addition to increasing stability and translation efficiency of in vitro transcribed mRNA molecules^20,21^.

Next, we delivered between 0.5 µg to 2.5 µg of the modified MyoD-mRNA to 1.5 × 10^5^ MEFs using a lipofectamine-based transfection reagent in a dose-dependent manner. This trial manifested robust MYOD expression in 60–70% of cells, as early as 5 h post-transfection and regardless of the amount transfected, which was comparable to MYOD expression level in dox-treated Rep-MEFs (Fig. 2b, c). However, after 24 h this percentage was substantially reduced to approximately 20% MYOD^+^ cells, in addition to exhibiting decreased fluorescence intensity (Figs. 2b, c and S5a). These results illustrate the transient nature of mRNA-mediated MyoD overexpression and suggest a need for repeated mRNA transfections to attain a high level of protein expression over time. As such, we tested whether multiple MyoD-mRNA transfections of fibroblasts over a course of 4 days might enable myogenic transdifferentiation, and consequently documented by this treatment the formation of MYHC^+^ myotubes (Fig. 2d). This encouraging result prompted us to examine whether repeated delivery of synthetic MyoD-mRNA in concert with F/R/C supplementation might promote the generation of PAX7^+^ iMPCs over time (Fig. 2e). In accordance with the transdifferentiation trial, treating *Pax7-CreERT2; R26-LSL-ntdTomato* MEFs with 1ug or 2ug of MyoD-mRNA over 4–5 days in concert with continuous F/R/C administration resulted in the formation of ntdTOMATO^+^/PAX7^+^ cells at day 10–15, which exhibited iMPC-like morphology (Figs. 2e, f and S5b). These iMPC-like colonies could be manually picked and propagated in the presence of F/R/C supplementation, giving rise to proliferative and heterogeneous iMPC-like clones which expressed a cohort of canonical myogenic markers including PAX7, MYOD, MYOG, and MYHC for at least 6 passages (Fig. S5c, d and Movie S1). Next, we trialed the production of iMPCs from MEFs harboring a *Pax7-nGFP* reporter using MyoD-mRNA and F/R/C. We successfully produced Pax7-nGFP^+^ iMPC colonies which remarkably expressed between 55.3% to 98.3% of Pax7-nGFP^+^ cells at passage 2–3 (Fig. S5e, f). Based on these results, we conclude that MyoD-mRNA in conjunction with F/R/C treatment can produce integration-free iMPCs.

As a following step, we sought to decipher whether we can detect myogenic stem, progenitor and differentiated cell populations in a transgene-free iMPC clone at passage 5 using single-cell RNA-sequencing (scRNA-seq). By means of this analysis, we deconstructed an iMPC clone into eight distinct cell clusters, four of which expressed *Pax7*, thus representing myogenic stem and progenitor cells (Pax7_PGs) (Fig. 2g–i). In addition, two cell clusters co-expressed *Myod1*, *Sox8* and *Dll1*, thus representing committed progenitors (comm_PGs), yet were separated by proliferation markers (Fig. 2g–i). Finally, two cell clusters expressed *Myh1*, *Tnnt3* and *Tnnc2*, representing differentiated skeletal muscle cells (diff_SMCs); however, separated by different expression of *Myod1* and *Myog* (Fig. 2g–i). Namely, the four *Pax7*^+^ cell populations were annotated based on a unique gene expression indicative of each cell population. For example, one *Pax7*^+^ cell population (Pax7_PGs-1) was annotated based on high expression of proliferation markers (*Ube2c, Mki67*) and *Myod1*, whereas the second *Pax7*^+^ cell population (Pax7_PGs-2) did not exhibit a high level of these genes (Figs. 2g–i, S6a, b). Additionally, another *Pax7*^+^ cell population (Pax7_PGs-4) expressed connective tissue-like cell markers (*Bgn*, *Pdgfrb*), in accordance with our previous observation that iMPCs can give rise to a connective tissue-like cell fate^41^ (Fig. 2g, h). Surprisingly, the remaining *Pax7*^+^ cell population (Pax7_PGs-3) demonstrated expression of genes indicative of satellite cells in vivo, including *Apold1*^53^, *Apoe*^53,54^*, CD9*^55^*, Cebpb*^56^ and most notably absence of *Cdkn1c*, a myoblast marker reportedly undetectable in muscle stem cells^57^ (Figs. 2g–i and S6a–c). We next performed an Over Representation Analysis (ORA) using the KEGG pathway database and documented enriched annotations for each *Pax7*^+^ cell population, corroborating their molecular distinction (Fig. S6d, e). Last, we performed an RNA velocity analysis which documented a myogenic differentiation program emanating from cycling and non-cycling *Pax7*^+^ progenitor cells that gave rise to *Sox8*^+^/*Myod1*^+^ committed progenitors and *MyHC*^+^ differentiated muscle cells (Fig. 2g–j). Intriguingly, we observed a branching point projecting from Pax7_PGs-1 and 2 to Pax7_PGs-3 (Fig. 2j). This observation cautiously suggests that a subset of the cycling Pax7^+^ iMPCs can de-differentiate into a population expressing muscle stem cell markers that are enriched in satellite cells in vivo. In summary, we devised a synthetic MyoD-mRNA delivery system that in conjunction with F/R/C supplementation gave rise to transgene-free iMPCs. These myogenic cultures can be expanded extensively in vitro, faithfully recapitulating a skeletal muscle differentiation program.

### Facilitating iMPC generation via MyoD-mRNA and enhanced small molecule cocktails

**Fig. 3:**
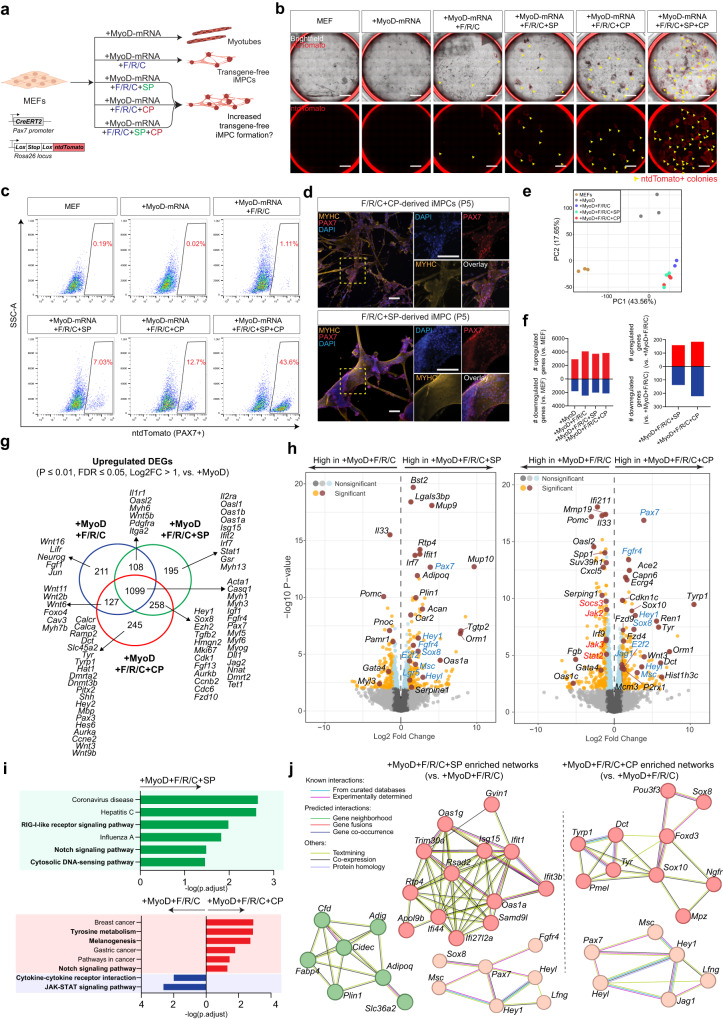
Enhanced iMPC production using synthetic MyoD-mRNA and small molecules. **a** A schematic showing experimental design. **b** Representative whole-well brightfield and fluorescence images demonstrating emerging PAX7^+^ colonies at day 14 of reprogramming of *Pax7-CreERT2; R26-LSL-ntdTomato* MEFs. Colonies are highlighted with yellow arrowheads. Cells were transfected with 2 μg MyoD-mRNA during the first 4 days. Scale bar, 5 mm. **c** FACS plots showing percentages of ntdTomato^+^ cells at day 14 of reprogramming of *Pax7-CreERT2; R26-LSL-ntdTomato* MEFs. Cells were transfected with 2 μg MyoD-mRNA on the first 4 days of reprogramming and labeled with 4-OHT two days prior to analysis. **d** Representative immunofluorescence images for the indicated markers in stable transgene-free iMPC clones reprogrammed and kept in F/R/C + CP or F/R/C + SP at passage 5. Scale bars, 100 μm. **e** PCA based on bulk RNA-seq data for the indicated conditions at day 10 of reprogramming with MyoD-mRNA and small molecules. *N* = 3, each dot represents a different cell line. **f** Number of differentially expressed genes (DEGs) for the indicated conditions relative to parental MEFs (left) or the “+MyoD+F/R/C” (right) condition at day 10 of reprogramming. Significantly upregulated or downregulated genes were determined using |log2FC | > 1 and *p*-value ≤ 0.01. **g** Venn diagram showing the overlap of upregulated genes across all the indicated conditions versus the “+MyoD” condition. Only significantly upregulated protein-coding genes are shown. A cut-off of log2FC > 1, *p*-value ≤ 0.01, FDR ≤ 0.05 was used. **h** Volcano plots showing DEGs for the indicated conditions at day 10 of reprogramming. Significance was calculated using |log2FC | > 1 and *p*-value ≤ 0.01. JAK-STAT signaling pathway associated genes are highlighted in red, whereas satellite cell-related genes are highlighted in blue. **i** Over-representation analysis (ORA) using the KEGG database for the indicated conditions. Only significant pathways are shown (p.adjust ≤ 0.05). **j** Gene networks for the indicated comparisons based on the STRING database. Only significantly upregulated DEGs (log2FC > 1, *p*-value ≤ 0.01) were used. Networks were clustered in an unbiased manner by Markov Cluster Algorithm (MCL) inflation parameter 3.0 (F/R/C + SP) or 2.4 (F/R/C + CP).

As the next objective, we set out to determine whether combining MyoD-mRNA+F/R/C with either SP, CP or SP + CP treatment might enable a more efficient derivation of PAX7^+^ iMPCs. To investigate this possibility, we optimized the transfection and reprogramming regimen utilizing *Pax7-CreERT2; R26-LSL-ntdTomato* fibroblasts subjected to MyoD-mRNA in concert with either F/R/C, F/R/C + SP, F/R/C + CP or F/R/C + SP + CP treatment (Fig. 3a). At day 14 of reprogramming, we detected around 1% ntdTOMATO^+^/PAX7^+^ iMPCs with F/R/C treatment and 7% and 13% with either the addition of SP or CP, respectively (Fig. 3b, c). Notably, the addition of both inhibitors significantly increased the number of PAX7^+^ cells to around 44%, and many more colonies appeared in the culture dish (Fig. 3b, c). Moreover, in a trial using refractory fibroblasts that did not reprogram into PAX7^+^ iMPCs via conventional MyoD-mRNA+F/R/C treatment, we could produce PAX7^+^ iMPCs with the addition of SP, CP, and more efficiently when using both inhibitors (Fig. S7a–d). Additionally, we confirmed these results using fibroblasts carrying the *Pax7-nGFP* genetic reporter (Fig. S7e, f). Interestingly, and in contrast to our prior observation using the lentivirus reprogramming system (Fig. 1), we documented increased formation of myotubes and fusion index with the addition of the two inhibitors by day 10 in iMPC medium, highlighting potential differences between the two MyoD delivery systems (Fig. S7g, h). Importantly, transgene-free iMPCs produced via F/R/C + CP could be propagated, expressing stem and differentiated cell markers (Figs. 3d and S7i). In support of this observation, scRNA-seq analysis of an iMPC clone generated with MyoD-mRNA+F/R/C + CP and maintained in F/R/C + CP condition revealed similar cell populations to iMPCs generated with MyoD-mRNA+F/R/C and maintained in F/R/C (Fig. S8a–i). This analysis also surprisingly uncovered a very small cell population expressing melanocyte markers such as *Pax3* and *Tyrp1* (Fig. S8a–c). In contrast, we noted that F/R/C + SP medium proved challenging to propagate iMPCs long-term, most likely due to extensive fusion and loss of proliferative potential over time (Figs. 3d and S7i).

To gain further molecular insights into the genes and pathways that might be altered following the suppression of the JNK or JAK/STAT pathways during iMPC reprogramming, we performed global transcriptome analysis. To this end, we subjected to bulk RNA-seq analysis 3 different MEF lines that have been treated for 10 days with either MyoD, MyoD + F/R/C, MyoD + F/R/C + SP and MyoD + F/R/C + CP (Fig. S9a). A PCA and a correlation matrix analysis separated MEFs, MEFs + MyoD and MEFs + MyoD + F/R/C into three distinct clusters, with SP or CP treated cells clustering in association with the MyoD + F/R/C condition (Figs. 3e and S9b, c). Additionally, in comparison to parental MEFs at day 10 of reprogramming between 3000 and 4000 genes were upregulated and 1500–2500 were downregulated for all tested conditions, emphasizing the extensive transcriptional changes associated with MyoD overexpression in fibroblasts; however, the addition of SP or CP only altered the expression of 150–200 genes in comparison to the MyoD + F/R/C condition (Fig. 3f).

Next, we compared MEFs treated with MyoD-mRNA+F/R/C to the same treatment with the addition of SP or CP at day 10 of reprogramming. First and as expected, a comparison of MyoD + F/R/C vs. MyoD revealed a notable group of upregulated satellite cell and differentiation markers unique to the F/R/C treatment, in addition to enrichment in skeletal muscle-related GO terms (Fig. S9d, e). Further, we noticed that in comparison to the F/R/C condition, several canonical satellite cell markers, including *Pax7*, *Msc*, *Fgfr4* and *Sox8*, were substantially higher under the F/R/C + SP and F/R/C + CP conditions; however, expression of differentiation genes such as *Myog*, *Myf6* and *Myh1* was unaltered (Fig. S9f). We then compared significantly enriched genes in each condition relative to the MyoD-mRNA condition, and identified shared or uniquely expressed markers (Fig. 3g). Most notably, whereas several satellite cell markers were upregulated in all conditions vs. MyoD alone (*Pax7*, *Myf5*, *Dmrt2*), other satellite cell markers were more enriched in one or two conditions including *Calcr, Heyl*, and *Lgr5* (CP enriched) or *Sox8* and *Hey1* (SP or CP enriched) (Figs. 3g and S9g). Accordingly, a volcano plot representation for differentially expressed genes revealed that several of the most upregulated genes in SP- or CP-treated cells in comparison to MyoD + F/R/C include satellite cell markers such as *Pax7*, *Fgfr4* and *Hey1* (Fig. 3h). Last, a functional enrichment analysis based on the KEGG and STRING databases for SP and CP treatment vs. conventional F/R/C treatment revealed enrichment for Notch associated genes, a signaling pathway which is highly enriched in satellite cells in vivo (Figs. 3i, j and S10a)^58^. In respect to downregulated genes, we documented reduction in immune system-related markers and pathways upon CP treatment (*Ifi44, Irf7, Irf9*) (Fig. S10a, b). We then employed additional functional annotation webtools and confirmed largely overlapping enriched terms for each condition, including reduction in JAK/STAT (*Jak2, Jak3, Stat2, Socs3*) or JNK (*Jun, Junb, Fos*) pathway-associated genes (Fig. S10a, b). In conclusion, we demonstrate that suppression of the JNK or JAK/STAT pathways by SP or CP administration in conjunction with MyoD-mRNA+F/R/C substantially increases iMPC derivation, and is characterized by an upregulation of several satellite cell-associated genes and pathways.

### Transgene-free iMPCs robustly restore dystrophin expression in muscles of DMD mice

**Fig. 4:**
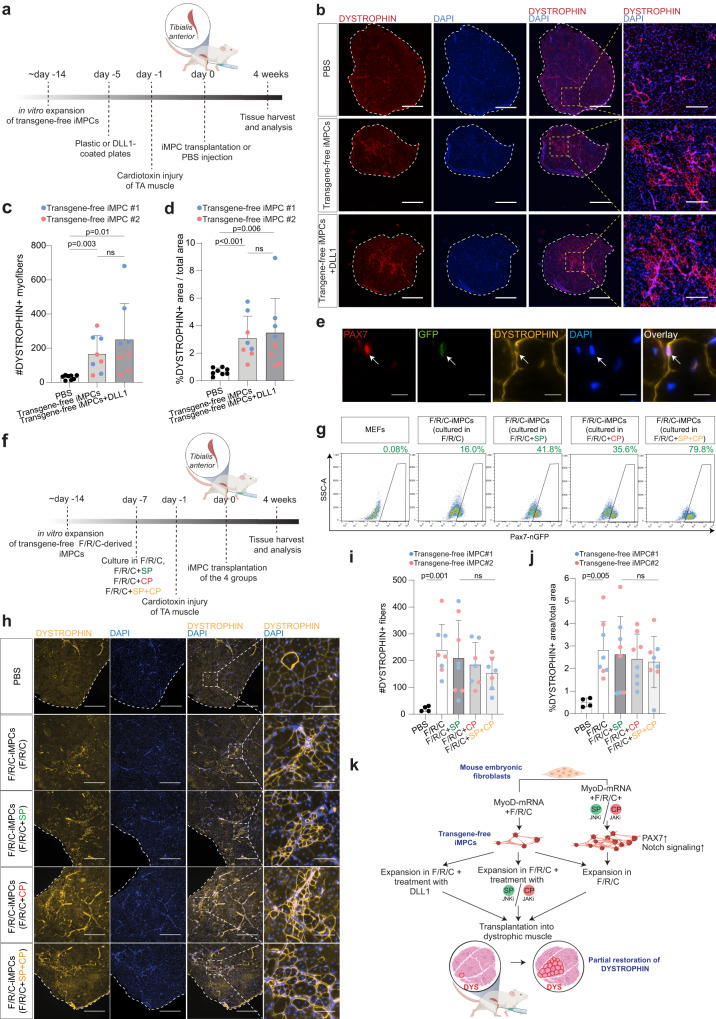
Transgene-free iMPCs restore DYSTROPHIN expression in DMD mice. **a** A schematic illustrating transplantation strategy of transgene-free iMPCs into dystrophic *Tibialis Anterior* (TA) muscles of DMD mice. **b** Representative immunofluorescence images of DYSTROPHIN in TA muscle cross-sections at 4 weeks post iMPC transplantation. PBS injection was used as a negative control. Scale bar, 1 mm; scale bar inlay, 200 μm. **c** Quantification of DYSTROPHIN^+^ fibers per cross-section in TA muscles engrafted with PBS or iMPCs treated with or without DLL1. Data are shown as mean ± SD. *N* = 2 iMPC lines, each transplanted four independent times into TA muscles as indicated by color coding. Significance was determined by two-tailed unpaired *t* tests. **d** Percentage of DYSTROPHIN^+^ area as calculated per total TA area. Related to (**b**). **e** Immunofluorescence image depicting a rare donor-derived PAX7-nGFP^+^ cell within a restored DYSTROPHIN^+^ area in a transplanted TA. Scale bar, 10 μm. **f** A schematic illustrating transplantation strategy of transgene-free iMPCs treated with SP, CP or SP + CP for 7 days prior to engraftment into TA muscles of DMD mice. **g** FACS analysis of an iMPC clone subjected to the indicated conditions for 10 days. **h** Representative immunofluorescence images of DYSTROPHIN in TA muscles 4 weeks after transplantation. Scale bar, 500 μm; scale bar inlay, 100 μm. **i** Quantification of DYSTROPHIN^+^ fibers per TA muscle cross-section engrafted with PBS or iMPCs treated with the indicated small molecules. Data are shown as mean ± SD. *N* = 2 iMPC lines, each transplanted four independent times into TA muscles as indicated by color coding. Significance was determined by two-tailed unpaired *t* tests. **j** Percentage of DYSTROPHIN^+^ area as calculated per total TA area. Related to (**h**). **k** A model summarizing the findings of this study.

We previously reported that MyoD lentivirus-derived iMPCs can engraft and efficiently restore dystrophin expression in limb muscles of DMD mice^40,59^. As such, we next set out to explore to what extent transgene-free iMPCs can contribute to muscle regeneration in dystrophic mice in vivo. To address this question, we opted to inject two transgene-free *Pax7-nGFP* iMPC clones into cardiotoxin pre-injured *Tibialis anterior* (TA) muscles of immunodeficient and dystrophic *Prkdc*^*scid*^*; Dmd*^*mdx*^ mice, seeking to establish whether engrafted myogenic cells can fuse and restore dystrophin expression in vivo (Fig. 4a). In a previous study, we determined that culturing iMPCs on the Notch ligand Dll1 enabled a more homogenous expansion of Pax7^+^ iMPCs^41^. We therefore decided to investigate whether treatment with Dll1 may augment the transplantation potential of F/R/C-derived transgene-free *Pax7-nGFP* iMPCs in vivo (Fig. 4a). This treatment preferentially precipitated a higher number of mononucleated Pax7-nGFP^+^ cells and less multinucleated myotubes (Fig. S11a, b). One-month post-transplantation, TA muscles were harvested, fixed and stained for DYSTROPHIN expression. We recorded a robust increase in the number of DYSTROPHIN^+^ myofibers following transplantation of iMPCs in comparison to PBS control, ranging between 150–300 myofibers in the majority of muscle sections (Fig. 4b, c). Notably, a statistically significant difference in respect to DYSTROPHIN restoration was not documented between Dll1-treated and non-treated iMPCs, albeit several Dll1-treated clones precipitated a substantially higher number of DYSTROPHIN restored myofibers, reaching up to 600 DYSTROPHIN^+^ myofibers and representing almost 10% of the total muscle area in one muscle section (Figs. 4b–d and S11c). Moreover, in several sections we could detect rare donor-derived Pax7-nGFP^+^ cells in association with DYSTROPHIN^+^ myofibers, indicating the contribution of iMPCs to the satellite cell reservoir (Fig. 4e). Together, we conclude that transgene-free iMPCs are fusion-competent and can efficiently contribute myonuclei to dystrophic myofibers, resulting in dystrophin restoration. In accordance with a previous report for satellite cells^60^ and excluding a few outliers, culturing iMPCs on Dll1-coated plates prior to engraftment did not manifest a statistically significant increase in the number of DYSTROPHIN positive myofibers.

Akin to Dll1 administration, treatment with SP or CP increases the number of PAX7^+^ cells in iMPCs, thus raising the possibility that these compounds might enhance the engraftment potential of established F/R/C-derived transgene-free iMPCs. To test this hypothesis, we treated two transgene-free *Pax7-nGFP* iMPC clones with either SP, CP or SP + CP treatment for 7 days prior to intramuscular transplantation into TA muscles of *Prkdc*^*scid*^*; Dmd*^*mdx*^ mice (Fig. 4f). Of note, we assessed the number of PAX7^+^ iMPCs following treatment and confirmed presence of PAX7^+^ cells under the F/R/C condition (16.0%) which increased with either SP, CP or SP + CP treatment (41.8%, 35.6%, and 79.8%, respectively), in addition to demonstrating an increase in fluorescence intensity (Figs. 4g and S11d). One month post-transplantation, we harvested and stained the muscles for DYSTROPHIN expression. Under all examined conditions, we could detect around 150–250 DYSTROPHIN^+^ myofibers and no appreciable level of cell migration away from the injection site (Fig. 4h, i). However, the number of DYSTROPHIN^+^ myofibers or restored area was similar for all examined conditions (Fig. 4i, j). This result suggests that increasing the number of transplanted PAX7^+^ iMPCs via SP, CP or dual SP + CP treatment does not result in a higher number of restored DYSTROPHIN^+^ myofibers. This observation is in accordance with our prior observation that pre-treatment with Dll1, which moderately increased the number of PAX7^+^ cells in iMPCs, did not yield an increase in restored DYSTROPHIN^+^ myofibers.

## Discussion

In this study, we report on a robust method to convert fibroblasts into muscle stem cells utilizing synthetic MyoD-mRNA and small molecule treatment. Specifically, we identified the JNK and JAK/STAT pathways as molecular roadblocks for iMPC formation, and demonstrated that suppression of these pathways facilitated reprogramming into PAX7^+^ iMPCs. As a second objective, we employed repeated MyoD-mRNA transfections in concert with F/R/C supplementation to produce transgene-free iMPCs. Further, by using scRNA-seq analysis, we dissected the various cell populations comprising stable transgene-free iMPC clones and identified a unique *Pax7*^+^ stem cell subpopulation which shares common denominators with satellite cells in vivo. Third, by combining the two approaches, we demonstrated an enhanced method to produce transgene-free iMPCs, further dissecting at the transcriptomic level genetic changes associated with suppression of the JNK and JAK/STAT pathways during reprogramming. Last, we report that transgene-free iMPCs can robustly engraft limb muscles of DMD mice, efficiently restoring dystrophin expression in approximately 3–10% of myofibers and contributing cells to the muscle stem cell pool, thereby unveiling potential utility for cell-based therapies. However, pre-treatment with Dll1, SP, CP or SP + CP did not enhance the in vivo engraftment capacities of transgene-free iMPCs (Fig. 4k).

MyoD is the most widely researched myogenic regulatory factor, and has been extensively studied in respect to its capacity to convert somatic cells into skeletal muscle cells^2,43–48,61–66^. These studies often utilized viral vectors to overexpress MyoD due to robust gene expression emanating from ubiquitous strong promoters. Recent works have also reported utilizing modified MyoD-mRNA to achieve transdifferentiation or PSC-differentiation into muscle cells^24,27,29,30,33^. It will be of interest to assess whether reprogramming via MyoD-mRNA might be more advantageous for switching cell fate in comparison to viral vector-based transdifferentiation, which reportedly generates only partially reprogrammed cells^42,67^. In support of this idea, studies on the production of iPSCs by mRNA delivery reported global gene expression that was more reminiscent of embryonic stem cells in comparison to viral vector-derived iPSCs^24^. Moreover, excision of viral vectors from iPSCs reportedly rendered them more akin to embryonic stem cells^68^. In contrast, a comprehensive study which inspected a variety of non-integrating iPSC reprogramming methods recorded no major differences between the various approaches, albeit mRNA-based delivery of transcription factors was noted for its rapidness, safety, and efficiency^69^. Last, it will be of interest to assess whether delivery of MyoD-mRNA can induce lineage conversion in vivo, as recent studies employed mRNA molecules to redirect cell fate for therapeutic purposes in situ^70–73^.

Our study identified two inhibitors that enhance PAX7^+^ iMPC production. The small molecule SP600125 is a selective inhibitor of JNK1-3, which belong to the MAPK family^74^. Upon activation, the downstream target of JNK, c-Jun, proceeds to form dimeric complexes called activating-protein 1 (AP-1) in concert with Fos family or ATF-related proteins^75^. To date, the role of JNK signaling and AP-1 during myogenesis remains controversial, supporting both inhibitory and promoting effects on myogenic differentiation^75–79^. As an example, a recent report implicated a role for MAPK/JNK in concert with MAPK/ERK signaling in promoting myogenesis via upregulation of MYOD and PAX7 proteins in a dystrophic mouse model^80^. In that model, JNK pathway upregulation was associated with an increase in Pax7 expression, highlighting its diverse role in comparison to this study, in which JNK downregulation promoted Pax7 upregulation during iMPC derivation. Additionally, JNK signaling is known to integrate stress-related signals such as reactive oxygen species (ROS) to activate apoptosis^81^. We previously documented that reprogramming of MEFs into iMPCs is associated with a metabolic switch from glycolysis to oxidative phosphorylation, which may impede cell survival due to increased ROS production^41^. Therefore, SP treatment might assist cell survival during iMPC reprogramming by downregulation of JNK signaling as a consequence of ROS production. Furthermore, our results reveal that in the context of iMPC reprogramming suppression of the JNK pathway enhances the derivation of Pax7^+^ muscle stem cells and increases Notch signaling, however, the precise mode of action and molecular mechanisms remain to be explored.

The JAK/STAT signaling pathway is a central regulator of inflammation and other cellular processes^81^. Delivery of exogenous mRNA is associated with upregulation of pro-inflammatory responses which can drastically impede the use of this technology^20,21^. Our data show that inhibition of JAK/STAT signaling by CP690550 downregulated inflammatory and immune-related processes and as such might result in higher cell survival and increased number of PAX7^+^ iMPCs. Furthermore, inhibition of the JAK/STAT (i.e. JAK1-3) pathway has been well-established in the context of muscle regeneration^82,83^. Specifically, JAK/STAT signaling is increased in aged muscle stem cells, accounting for a reduced regeneration potential that is reversible with JAK inhibition^82^. Specifically, inhibition of JAK2 and its downstream adapter molecule STAT3 has been shown to increase the number of PAX7-expressing satellite cells, in addition to augmenting muscle stem cell engraftment in vivo^82,83^. We postulate that similar mechanism/s may also govern the increase in PAX7^+^ cells in CP-treated iMPCs in vitro. Accordingly, recent works have utilized small molecules to improve satellite cell proliferation and regeneration potential in vivo, with several studies utilizing the small molecules employed in this work including F^84^, F/R^85^ and JAK inhibitors^82,83,86^ to treat muscle pathologies. As such and given their administration in this study did not increase the transplantation potential of iMPCs in a dystrophic mouse model, it will be of interest to assess whether systemic administration of SP or CP with F/R/C may augment a myogenic regeneration response in vivo, potentially proving beneficial to the remedy of muscle pathologies.

In summary, to the best of our knowledge this study represents the first transgene-free direct conversion of fibroblasts into muscle stem cells utilizing synthetic mRNA encoding for a transcription factor. As such, implications may also be relevant to other transcription factor-based lineage conversion systems in the form of fibroblast conversion into neural, cardiac, or hepatic progenitors, which to date were mostly documented using viral vectors^87–91^. Due to a multitude of recent challenges associated with viral vectors and gene therapy trials^92^, directly reprogrammed cells may also encounter comparable hurdles. We envision that a synthetic mRNA-based delivery of transcription factors may continue to evolve as an alternative and safer approach to reprogram cell fate for regenerative medicine purposes.

## Methods

### Animals

The following mouse strains were obtained from Jackson Laboratories and used in this study: *B6.Cg-Pax7*^*tm1(cre/ERT2)Gaka*^/*J* (Jax strain number: 017763), *B6.Cg-Gt(ROSA)26Sor*^tm75.1(CAG-tdTomato*)Hze^/*J* (Jax strain number: 025106), *B10ScSn.Cg-Prkdc*^*scid*^ *Dmd*^*mdx*^/*J* (Jax strain number: 018018). The strains *B6.Cg-Pax7*^*tm1(cre/ERT2)Gaka*^/*J* and *B6.Cg-Gt(ROSA)26Sor*^tm75.1(CAG-tdTomato*)Hze^/*J* were crossed to homozygosity to obtain *Pax7-CreERT2*; *R26-LSL-ntdTomato* reporter MEF lines. The *Pax7-nGFP* reporter MEF lines were obtained from crossing homozygous *Tg*:*Pax7-nGFP/C57BL6*;*DBA2* mice^51^. All mice used in this study were housed in specific-pathogen-free (SPF)-like conditions according to the Swiss Federal Law on Animal Protection and approved by the Cantonal Animal Welfare Committee (license numbers ZH108/2018, ZH177/2018 and ZH002/2022).

### MEF culture

Mouse embryonic fibroblasts were isolated at E13.5 and single embryos were removed from uteri and henceforth treated as separate cell lines. Head bud, internal organs and intestinal tissue were separated from the torso and the remaining embryonic tissue was dissociated and minced into a uniform slur using scalpels. The tissue was further digested in 0.25% Trypsin (Thermo Fisher Scientific, Cat. #25200056) for 10 min at 37 °C, collected in MEF medium, centrifuged at 500 × *g* and plated for culture. At roughly 80% confluency, P0 MEFs were trypsinized, strained through a 100 μm cell strainer and either frozen or expanded for further experiments. Lentiviral transduction was performed in passage 1. For reprogramming experiments, MEFs at passages 1–3 were used. All cell lines were cultured at 37 °C in 20% O_2_ for expansion and reprogramming. All cell lines were individually tested for mycoplasma contamination prior to experimentation using the MycoAlert Mycoplasma Detection Kit (Lonza, Cat.# LT07-118).

### Lentivirus production and transduction

Lentiviruses were produced in HEK-293T cells and precipitated in PEG-it Virus Precipitation Solution (System Biosciences, Cat. #LV825A-1-SBI). Briefly, HEK-293T cells were cultured to 60–70% confluency in high glucose DMEM (Thermo Fisher Scientific, Cat. #41966029) containing 10% fetal calf serum (FCS) (Thermo Fisher Scientific, Cat. #10270106) and 1% Pen/Strep (Thermo Fisher Scientific, Cat. #15-140-122). Packaging (16.5 µg), envelope (11 µg) and target (22 µg) DNA vectors were mixed with NaCl solution (150 mM) to a volume of 1 ml (see supplementary table 5 for more details about plasmids used in this study). Then, 1 ml of polyethylenimine (PEI) (Chemie Brunschwig AG, Cat. #POL23966-1) was added and the mixture was transfected into HEK-293T cells. All indicated amounts and volumes were used in a 15 cm cell culture plate format and medium was replaced every 24 h. Lentivector-containing medium (supernatant) was collected at 48 h and 72 h post-transfection and stored at 4 °C. Next, the supernatant was filtered through a 0.45 µm syringe filter and 0.25 ml of cooled PEG-it Virus Precipitation Solution was added per 1 ml of lentiviral vector-containing supernatant and stored overnight at 4 °C. This mixture was centrifuged at 1500 × *g* for 30 min at 4 °C the following day. The supernatant was then removed, and precipitated lentivirus particles were resuspended in PBS containing 25 mM HEPES buffer (Thermo Fisher Scientific, Cat. #15630106) in 1/10 to 1/100 of the original volume and stored in 20 µl aliquots at −80 °C until further use. MEFs were transduced at passage 1 (60–70% confluency) with one aliquot of frozen lentiviral vectors that was mixed with Polybrene transfection reagent (5 µg/ml) (Sigma-Aldrich, Cat. #TR-1003-G). Alternatively, to produce MEF lines with high reprogramming efficiencies (Rep-MEFs #4-6), we used ready-made concentrated lentiviral vectors encoding for MyoD or rtTA (produced by VectorBuilder), with transducing units ranging between 2.7 × 10^8^ and 1.0 × 10^9^ TU/ml. At 24 h post-transduction, cells were washed in PBS and grown to confluency in MEF medium. Cells were then expanded and sequentially subjected to an antibiotic selection for the two lentiviral cassettes with puromycin (1 µg/ml, 3 days) (Thermo Fisher Scientific, Cat. #A1113803) and G-418 (1 mg/ml, 4 days) (Sigma-Aldrich, Cat. #4727878001).

### MEF to iMPC reprogramming utilizing lentiviruses

Reprogrammable MEFs were plated at a density of 100,000–150,000 cells per well in a six-well plate one day prior to reprogramming in an equal mix of 1:1 MEF and iMPC media. See supplementary table 2 for more information about media used in this study. To induce reprogramming, cells were washed in PBS and placed in iMPC medium containing doxycycline (2 µg/ml) (Sigma-Aldrich, Cat. #D9891) and respective small molecules. See supplementary table 3 for more details about small molecules used in this study. During reprogramming, cell medium, doxycycline and small molecules were replaced every other day.

### In vitro transcription of mRNA

MyoD-mRNA was largely produced as described before^93^. As DNA template for in vitro transcription, we used a plasmid containing the MyoD coding sequence flanked at the 5′ end by a T7 promoter sequence followed by a 5′UTR sequence, and at the 3′ end by the 3′UTR of the alpha 1 globin gene (Hba1, ENSMUSG00000069919) and a 30 bp poly A tail (see supplementary table 5 for more details). The plasmid was linearized using the restriction enzyme FastDigest LguI (SapI) (Thermo Fisher Scientific, Cat. #FD1934) and purified using the QIAquick PCR purification kit (Qiagen, Cat. #28104) according to the manufacturer’s protocol. Using tail-PCR, the digested plasmid was supplemented with a 120 bp poly-A tail using the following primers: Forward primer: 5′-TAT CAC GAG GCC CTT TCG TCT AAT ACG-3. Reverse primer: 5′-TTT TTT TTT TTT TTT TTT TTT TTT TTT TTT TTT TTT TTT TTT TTT TTT TTT TTT TTT TTT TTT TTT TTT TTT TTT TTT TTT TTT TTT TTT TTT TTT TTT TTT TTT TTT TTT TTT TTT TTT TCA AAG ACC AAG AGG TAC AGG TGC AAG-3'. Tail-PCR was performed using Phusion High Fidelity DNA polymerase (NEB, Cat. #M0530) according to the manufacturer’s protocol and 1 ng of linearized DNA template. PCR was performed as follows: denaturation @98 °C for 30 s, amplification [@98 °C for 10 s, @68 °C for 30 s, @72 °C for 45 s (30 cycles)], and final extension @72 °C for 600 s. For in vitro transcription, we used the MEGAscript T7 Transcription Kit (Thermo Fisher, Cat. #AMB13345). Reactions were set up in 40 µl total volume per tube using a nucleotide mix consisting of 6 mM Anti-Reverse Cap Analog (ARCA) (NEB, Cat. #S1411S), 1.5 mM GTP (Megascript T7 kit), 7.5 mM ATP (Megascript T7 kit), 7.5 mM 5-Methylcytidine-5′-Triphosphate (Trilink, Cat. #N-1014-1) and Pseudouridine-5′-Triphosphate (Trilink, Cat. #N-1019-1). We used 1.6 µg of tailed PCR template per reaction, 1 × T7 buffer and 1 × T7 enzyme mix in nuclease-free H_2_O. In vitro transcription reactions were incubated for 5 h at 37 °C and subsequently treated with 2 µl DNase (Megascript T7 kit) per reaction for 15 min at 37 °C. Next, the reactions were purified using the MEGAclear Transcription Clean-Up Kit (Thermo Fisher, Cat. #AM1908) according to the manufacturer’s protocol. Following purification, mRNA was treated with 2 µl of Antarctic phosphatase (NEB, Cat. #M0289S) per reaction for 1 h at 37 °C and as per the manufacturer’s instructions. Lastly, the reaction mix was purified again using the MEGAclear Transcription Clean-Up Kit. RNA quantity and quality were assessed using a TECAN plate reader. The mRNA concentration was adjusted to 0.1 µg/µl or 0.2 µg/µl using nuclease-free water and single-use aliquots of 1 µg or 2 ug were stored at −80 °C until further use.

### MEF to iMPC reprogramming utilizing synthetic MyoD-mRNA

For mRNA-based reprogramming, MEFs were maintained in antibiotic-free conditions during the duration of transfections. One day prior to reprogramming, MEFs were plated at a density of 120,000–150,000 cells per well in a 6-well plate format in iMPC medium. Successful reprogramming was achieved when cells reached 60–70% confluency after an overnight culture. Of note, we observed several times refractory MEF lines that were not reprogrammable using this method, likely due to senescence and loss of proliferation potential. On the day of reprogramming, cells were washed in PBS and placed in iMPC medium containing small molecules and in the presence of a recombinant B18R protein (200 ng/ml) (R&D systems, Cat. #8185-BR). After 1–2 h, transfections with MyoD-mRNA were performed using Lipofectamine MessengerMAX Transfection Reagent (Thermo Fisher Scientific, Cat. #LMRNA001) according to the manufacturer’s protocol. Per well of a six-well plate, 3.75 µl of Lipofectamine MessengerMAX reagent was mixed with Opti-MEM medium (Thermo Fisher Scientific, Cat. #31985062) to a final volume of 125 µL and incubated for 10 min at room temperature. Then, 1 or 2 µg of MyoD-mRNA was diluted in Opti-MEM medium to a final volume of 125 µL per one well of a six-well plate. Lipofectamine and mRNA dilutions were then mixed and incubated for 5 min at room temperature. Finally, the mixture was dispersed equally onto the cells in a drop-wise manner. For transfections in 12-well or 24-well formats all quantities were reduced by a factor of 2.5 or 5, respectively. At 24 h post-transfection, cells were gently washed in PBS, medium was replaced, and cells were immediately transfected again as described, for a total of 4–5 transfections. Reprogramming was successful when a stable balance between myotubes and mononucleated cells remained throughout the reprogramming period and when dense, contracting myogenic colonies started to form. The recombinant B18R protein was kept in medium for two more days after the last transfection to reduce potential RNA-mediated immunogenic cytotoxicity. About 10–14 days after initiation of reprogramming, iMPC-like clusters were split as a bulk to produce a stable line, or alternatively single myogenic colonies were picked and propagated to establish iMPC lines. To pick colonies, myogenic colonies were excised and detached from the culture plate using a pipet tip and placed in 0.25% Trypsin for 5 min at 37 °C before further culture in iMPC medium. Stable mRNA-derived iMPC clones were maintained in iMPC medium and small molecules and split in a 1:6 to 1:12 ratio upon confluency.

### DLL1 treatment of iMPCs

To test the effect of DLL1 on the engraftment potential of iMPCs, we cultured transgene-free iMPCs on DLL1-coated plates for 5 days prior to transplantation. To coat plates, we incubated plates with 1 µg/ml recombinant mouse DLL1 (R&D system, Cat. #5026-DL, diluted in PBS containing 0.1% BSA) overnight at 4 °C. On the next day, coated plates were pre-warmed at 37 °C, DLL1 was aspirated and iMPCs were placed directly on the coated wells upon splitting.

### Flow cytometry

Cells were detached using 0.25% Trypsin incubation for 5 min at 37 °C and washed twice in PBS, resuspended in FACS buffer (PBS + 2% FCS) and strained through a FACS tube containing cell strainer (35 µm filter). For analysis, we excluded doublets and dead cells using DAPI staining (1:1000) (Thermo Fisher Scientific, Cat. #62248). Around 10’000 events were recorded per sample unless otherwise stated. All samples were analyzed using a Sony SH800S Cell Sorter (Sony Biotechnology Inc.). Data analysis was performed using FlowJo Version 10.6.1.

### Cell counting experiments

Around 10,000 mononucleated iMPCs at P4, that have been derived and cultured in F/R/C, F/R/C + SP or F/R/C + CP were seeded onto 24-well plates and grown to confluency as per the indicated conditions. After 10 days, 10,000 iMPCs were reseeded in the same format and counted every 3 days. Cells were detached by trypsinization, spun down, resuspended in 1 ml medium, and counted using a Neubauer chamber.

### Immunofluorescence of cell lines

Cells were washed in PBS and fixed in 4% paraformaldehyde for 5 min. Next, cells were placed in a blocking solution (2% BSA and 0.5% Triton-X in PBS) for 30 min. Then, the cells were incubated with the primary antibodies for 60 min followed by two PBS washes. The cells were then incubated in secondary antibodies and DAPI (1:1000) for nuclear stain for 30 min. Finally, wells were PBS-washed again and covered with Prolong Gold Antifade Mountant (Thermo Fisher, Cat. #P36930). For co-staining of PAX7 and KI67, we performed overnight incubation at 4 °C with primary antibodies.

### Immunofluorescence of skeletal muscle sections

To assess for engraftment of transgene-free iMPCs, we stained cryo-sectioned iMPC-engrafted *tibialis anterior* dystrophic muscles for dystrophin expression. Sections (10 µm) were fixed in 4% paraformaldehyde for 5 min and washed twice in PBS. Slides were then subsequently blocked and permeabilized in blocking buffer (1% BSA, 0.2% Triton in PBS) for 15 min. Then, slides were incubated in anti-dystrophin primary antibody for 60 min before incubation with a secondary antibody and DAPI (1:1000) for 30 min. Slides were washed twice in PBS between incubations. Finally, we used Prolong Glass Antifade Mountant (Thermo Fisher, Cat. #P36980) to mount glass cover slips on stained sections. For the triple staining of PAX7, DYSTROPHIN and GFP, we added another blocking step for 1 h using mouse on mouse blocking reagent (Vector laboratories, Cat. #MKB-2213-1) to reduce unspecific binding and proceeded with overnight incubation at 4 °C with primary antibodies. See the antibody table (supplementary table 4) for more information about the antibodies used in this study.

### Intramuscular transplantation of iMPCs

To assess engraftment potential of transgene-free iMPCs, we transplanted stable transgene-free iMPC clones into pre-injured *tibialis anterior* (TA) muscles of 16–37 week old male immunodeficient *Prkdc*^*scid*^; *Dmd*^*mdx*^ mice. TAs were injured 1 day prior to cell transplantation by intramuscular injection of cardiotoxin (10 µM) (Latoxan Laboratory, #L8102) using a 29 gauge insulin syringe (BD, Cat. #324702). The iMPC clones were collected by trypsinization at confluency, or near confluency when contracting myotube networks were apparent alongside mononucleated cells in the culture dish. To this end, iMPC cultures were first washed in PBS and trypsinized for 5 min at 37 °C. Around 1 × 10^6^ cells (mononucleated, non-filtered) were manually counted and placed into individual Eppendorf tubes for each transplanted muscle. The iMPCs were then pelleted at 200 × *g*, the supernatant was removed, and cells were topped with 20 µl PBS and placed on ice. Prior to transplantation, cells were resuspended and injected using a 29 gauge insulin syringe (BD, Cat. #324702). Control conditions included equal volumes of cell-free PBS that has been injected into pre-injured TA muscles of *Prkdc*^*scid*^; *Dmd*^*mdx*^ mice.

### Muscle freezing and tissue sectioning

Four weeks post iMPC transplantation, TA muscles were harvested to assess engraftment potential of transplanted transgene-free iMPCs. Mice were euthanized and TAs were extracted and vertically mounted on 10% Tragacanth gum (Sigma-Aldrich, Cat. #G1128) fixed on a piece of wood cork. TA muscles were then immersed for 30 s in pre-cooled isopentane placed in liquid nitrogen. The tissue was then immersed in liquid nitrogen and stored at −80 °C until further use. Sectioning was performed using a cryostat (Leica, Cat. #CM1950) with a cross-section thickness of 10 µm. Each TA was mounted on 6–8 glass slides comprising 12–18 sections per slide (corresponding to roughly 60–100 µm muscle depth between sections). All harvested muscles were sectioned up to at least 50% of their respective length.

### Western blot

For protein isolation, cells were trypsinized, washed in PBS, spun down and resuspended thoroughly in 100 µl RIPA buffer (50 mM Tris base, 150 mM NaCl, 2 mM EDTA, 1% Triton-X, 0.1% SDS) containing 1x HALT protease inhibitor (Thermo Fisher Scientific, Cat. #87785). Samples were then centrifuged at 10,000 × *g* for 15 min at 4 °C and supernatant was taken. Protein content was quantified using the DC Protein Assay (Biorad, Cat. # 5000116) using BSA as protein standard. Samples were mixed in 1xLaemmli (Biorad, Cat. #1610747) containing 10% 2-Mercaptoethanol and boiled for 5 min at 95 °C. Samples were run on 4–20% Mini-PROTEAN TGX Stain-Free precast polyacrylamide protein gels (Biorad, Cat. #4568094). Equal amounts of proteins (15 μg) were loaded along PageRuler Plus Prestained Protein Ladder (ThermoFisher, Cat. #26619). Protein blotting was performed by employing the Trans-Blot Turbo Transfer System (Biorad, Cat. #1704156) using the mixed molecular weight setting. Blots were then blocked in TBS-T + 5% milk for 1 h at room temperature and incubated overnight in primary antibodies at 4 °C. The following day, blots were washed three times in TBS-T for 10 min and then incubated in HRP-linked secondary antibody for 1 h at room temperature. Next, blots were washed three times for 10 min in TBS-T and developed in Clarity Western ECL substrate (Biorad, Cat. #1705060) according to the manufacturer’s protocol. Blots were imaged using the Chemidoc imaging system (Biorad). Loading controls represent images of stain-free gels containing total protein. All incubation steps were performed on a shaker. TBS-T consisted of 50 mM Tris base, 154 mM NaCl and 0.1% Tween-20. See the antibody table (supplementary table 4) for further information about antibodies used in this study. All blots were derived from the same experiment and processed in parallel.

### Microscopy and image analysis

All microscopy imaging was performed using a Nikon ECLIPSE Ti2 microscope. Mean fluorescence intensity was quantified using the internal Nikon Ti2 microscope “JOBS” image quantification tool that records an average fluorescence intensity of all cells per image harboring the fluorophore of interest. All other quantifications, as wells as ROI selections and measurements for tissue immunofluorescence staining were performed using the multipoint and polygon selection tools using ImageJ software (Ver. 1.53e). Unstained or cells stained solely with secondary antibody served as a negative control for assessing background signal and adjusting image intensity. Fluorescence intensities of fluorophores were equalized for each experiment and for each magnification level (except for DAPI and MyHC staining). Fluorescence levels in Fig. 3D and S7I were adjusted individually due to differences in cell density. Whole well images were taken by whole well scanning and later resized for applicability using the ImageJ “Resize” plugin^94^. The fusion index was defined as the portion of all (MYHC^+^ DAPI^+^) cells /DAPI^+^ cells. For quantification of DYSTROPHIN^+^ fibers and DYSTROPHIN^+^ area in skeletal muscle sections, the TA cross-section with the highest number of DYSTROPHIN^+^ myofibers and area was selected for quantification of each condition.

### RT-qPCR

RNA was isolated using the RNeasy Mini Kit (Qiagen, Cat. #74104) as per the manufacturer’s protocol. RNA was quantified and quality-checked using TECAN plate reader. RNA was reverse transcribed using the High-Capacity cDNA Reverse Transcription Kit (Thermo Fisher Scientific, Cat. #4368813) as per the manufacturer’s protocol. Quantitative PCR was performed using the PrimeTime Gene Expression Master Mix (IDT, Cat. #1055771) for probe-based RT-qPCR. See supplementary table 1 for probe primer sequences. A 10 ng cDNA was used for each reaction and *Gapdh* was used as an internal endogenous control. Fold changes were calculated using the ΔΔCT method relative to the expression levels in MEFs. All qPCR reactions were performed using the QuantStudio5 qPCR machine (Thermo Fisher Scientific, Cat. #A34322).

### EdU analysis

To assess cell proliferation, EdU staining was carried out using the Click-iT EdU Alexa Fluor 647 Flow Cytometry Assay Kit (Thermo Fisher Scientific, Cat. #C10424) according to the manufacturer’s protocol. Briefly, 10 µM EdU was added to the cell culture medium and cells were incubated for 1.5 h at 37 °C. Cells were then briefly washed with PBS containing 1% BSA, harvested from tissue culture plates and fixed in 100 µl Click-iT fixative solution for 15 min. Fixed cells were then incubated in 100 µl Click-iT saponin-based permeabilization solution for 15 min, followed by incubation in 500 µl Click-iT reaction cocktail containing Alexa Fluor 647 azide for 30 min and washed with permeabilization solution. After staining, EdU-positive cells were FACS-analyzed.

### Bulk RNA-seq

For initial quality control, total RNA isolated using the RNeasy mini kit was applied to a Fragment Analyzer (Agilent, Santa Clara, California, USA) to measure the RNA integrity number (RIN). Samples with RIN above 8 were used for further library preparation which was conducted according to the TruSeq Stranded mRNA protocol (Illumina, Inc., California, USA). Briefly, poly-A enrichment was carried out for total RNA (100–1000 ng), followed by reverse-transcription of poly-A enriched RNA into cDNA. The cDNA was then ligated with TrueSeq adapters including unique dual indices (UDI) after fragmentation, end-repair and adenylation steps. Using PCR, cDNA containing TruSeq adapters on both ends was selectively amplified and further applied to a Fragment Analyzer to determine the quality and quantity of libraries. The average fragment size was approximately 360 bp. Last, 10 nM libraries diluted in Tris-Cl buffer (10 mM, pH 8.5) supplemented with 0.1% Tween-20 were used for sequencing on Novaseq 6000 (Illumina, Inc., California, USA) with single end reads.

### Bulk RNA-seq data analysis

The raw reads were first cleaned by removing adapter sequences and poly-x sequences (>9 nt used for detection) using fastp (Version 0.20.0)^95^. Sequence pseudo alignment of the resulting high-quality reads to the Mouse reference genome (build GRCm38.p6) and quantification of gene-level expression (gene model definition from GENCODE release 23) was carried out using Kallisto (Version 0.46.1)^96^. To detect differentially expressed genes we used the glm approach implemented in the software package DESeq2 (R version: 4.1.0, DESeq2 version: 1.34.0)^97^. Genes showing altered expression with adjusted Benjamini and Hochberg method, *p*-value ≤ 0.05 and log2 fold-change > 0.5 were considered to be differentially expressed. Over-representation analysis (ORA) was conducted based on differentially expressed genes with adjusted *p*-value ≤ 0.05 and log2 fold-change > 1. For detailed analysis criteria see respective figure legends. Cross-database enrichment analyses and protein network analyses were performed using the integrative webtools Enrichr^98^ and String-DB^99^, respectively.

### Single-cell RNA-seq

A transgene-free stable iMPC clone at passage 6 was collected by trypsinization from a cell culture plate. To remove cell debris and multi-nucleated myotubes present in iMPCs culture, collected cells were filtered through a 40 µm cell strainer (VWR, Cat. #734-0002). Filtered cells were then resuspended in PBS and used for cell counting. Cell viability was checked with Trypan blue (Sigma-Aldrich, Cat. #T8154) staining. Next, cells were diluted in PBS at 1000 cells/µl and used by a 10x Genomics platform. Chromium Next GEM Single cell 3’ v3.1 protocol was used according to the manufacturer’s instruction. In short, a Gel Bead-In Emulsions (GEM) was generated by loading cells in chromium Next GEM chip G targeting ~5000 cells in recovery. GEM was then incubated in a thermal cycler and cleaned with Dynabeads followed by cDNA amplification. Amplified cDNA was used for fragmentation followed by end repair and A-tailing. Next, adaptor ligation and index PCR were performed using single index plate T set A and double size selection was carried out using AMPure XP (Beckman Coulter, Cat. #A63881). The library was sequenced on Novaseq 6000 (Illumina, Inc, California, USA) with paired ends.

### Single-cell RNA-seq data analysis

CellRanger v7.0.0 pipeline^100^ was used for demultiplexing the samples, aligning raw reads against mouse reference genome assembly (build GRCm39), processing cell barcodes and counting unique molecular identifiers (UMIs). For F/R/C + CP-derived iMPCs, ambient RNA count was corrected with the R package SoupX v1.5^101^. The filtered feature-barcode count matrix for iMPC and SoupX corrected raw feature-barcode count matrix for F/R/C + CP- iMPCs were further analyzed using the Seurat v4.2.1 pipeline^102,103^. For quality control, cells with unique feature counts < 250 and > 4000 (F/R/C-iMPCs)/7000 (F/R/C + CP-iMPCs), mitochondrial gene counts > 15% and ribosomal gene counts > 40% were removed. The filtered data were log normalized and scaled. Principal component analysis (PCA) was performed on the scaled data using 2000 highly variable genes for dimensional reduction. Louvain algorithm^104^ was applied with a resolution of 0.5 to cluster the cells based on the first 30 principal components (PCs). Clustered cells were visualized in two-dimensional space using the uniform manifold approximation and projection (UMAP)^105^ of the same PCs. Anchor cells were determined using canonical correlation analysis method to integrate the samples together. Integrated data were scaled, clustered and visualized in two-dimensional space in a similar way as the individual samples. Wilcoxon rank-sum test with log2 fold-change > 0.25 and adjusted *p*-value < 0.01 was used to determine the cluster markers. In order to perform RNA velocity, raw reads were re-aligned to the Mouse reference genome (build GRCm38.p6) using STARSolo v2.7.8a^106^ running in the CB_UMI_Simple mode with multi-gene UMI filtering. Quantification of spliced and unspliced reads was performed by providing “Gene Velocyto” to the soloFeatures flag. Cell barcodes were then filtered to include only those used for the previous analyses. All count matrices (gene-level, spliced, and unspliced) were combined into a single SingleCellExperiment v1.18.0^107^ object. The R package scuttle v1.6.2^108^ was used for log normalization. The R package Velociraptor v1.6.0 was used as a wrapper around the Python package scvelo v0.2.4^109^ to perform the RNA velocity calculations in dynamical mode using the top 1000 highly variable genes and 50 nearest neighbors. Due to low feature counts, diff_SMCs2 was excluded from velocity analysis of iMPCs.

### Statistical analysis

Statistical analyses were carried out in GraphPad Prism (v9.2.0) or in R (v4) with RStudio (v4.2.0/2022.02.2) using appropriate statistical tests as indicated in respective figure legends.

### Reporting summary

Further information on research design is available in the Nature Research Reporting Summary linked to this article.

### Supplementary information

20230717_Supplementary_Information

supplementary video 1

20230710_nr-reporting-summary

## Data Availability

The bulk and single-cell RNA sequencing data generated as part of this study are available in gene expression omnibus (GEO) with the accession number GSE208064. Further data and protocols from this study can be requested from the corresponding author.
