# Supplementary material for: Transgene-free direct conversion of murine fibroblasts into functional muscle stem cells
Source: NPJ Regen Med. 2023 Aug 8;8:43. doi: 10.1038/s41536-023-00317-z (PMC10409758; doi:10.1038/s41536-023-00317-z)
Supplement: Supplementary file 1 — 20230717_Supplementary_Information [file 41536_2023_317_MOESM1_ESM.pdf]

Supplementary Figure 1

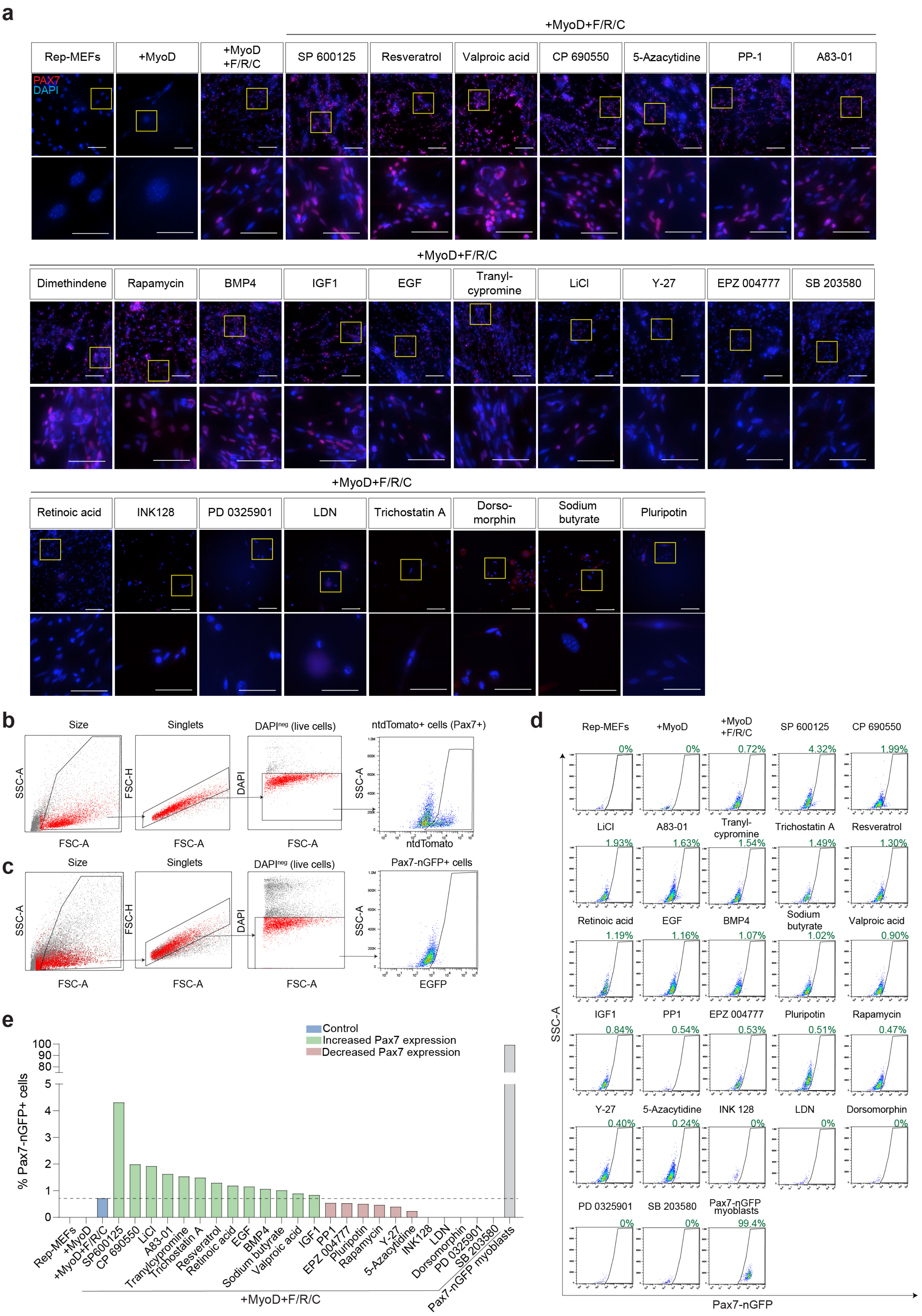

Supplementary Figure 1: Compound screen identifies small molecules that facilitate iMPC production

(a) Representative immunofluorescence images for PAX7 expression 14 days after initiation of reprogramming with the indicated conditions. Scale bar, 100μm; scale bar inlay, 50μm. **(b)** Representative FACS plots depicting gating strategy used in this study to detect ntdTomato^{+} cells. **(c)** Representative FACS plots depicting gating strategy used in this study to detect Pax7-nGFP^{+} cells. **(d)** FACS plots showing Pax7-nGFP expression in Rep-MEFs subjected to the indicated conditions for 14 days. **(e)** A graph showing quantification of (d).

Supplementary Figure 2

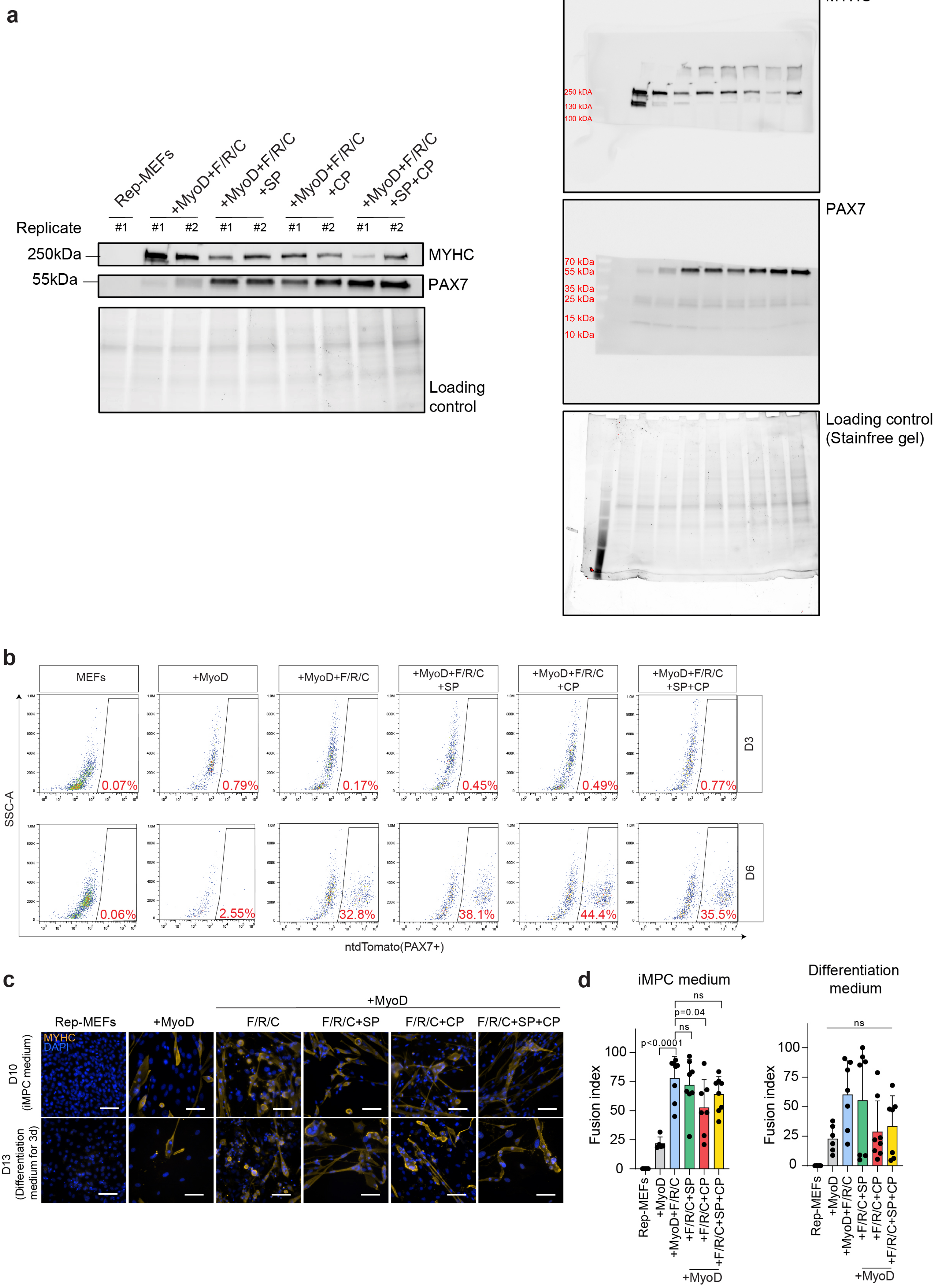

Supplementary Figure 2: Pax7 expression and fusion index analysis following SP or CP treatment

(a) Western blot analysis showing MYHC and PAX7 protein expression in MEFs subjected to the indicated conditions at day 10 of reprogramming. Stain-free gel is shown as protein loading control. The results for two different MEF lines are shown. Processed (left) and unprocessed (right) images are shown. **(b)** FACS-analysis of Pax7-CreERT2; R26-LSL-ntdTomato Rep-MEFs reprogrammed in the presence of the indicated conditions and days. 4-OHT was kept from day 0 of reprogramming. **(c)** Representative immunofluorescence images of Rep-MEFs at day 10 in iMPC medium or day 13 (10 days in iMPC medium, 3 days in differentiation medium) of a reprogramming course. Scale bar, 100µm. **(d)** Quantification of fusion index in the indicated reprogramming conditions. Per condition, 5-8 random fields of view were quantified. Data are shown as mean±SD**.** Significance was determined by ordinary one-way ANOVA using Dunnett's multiple comparisons test taking MyoD+F/R/C as control condition (ns=non-significant).

Suppelementary Figure 3

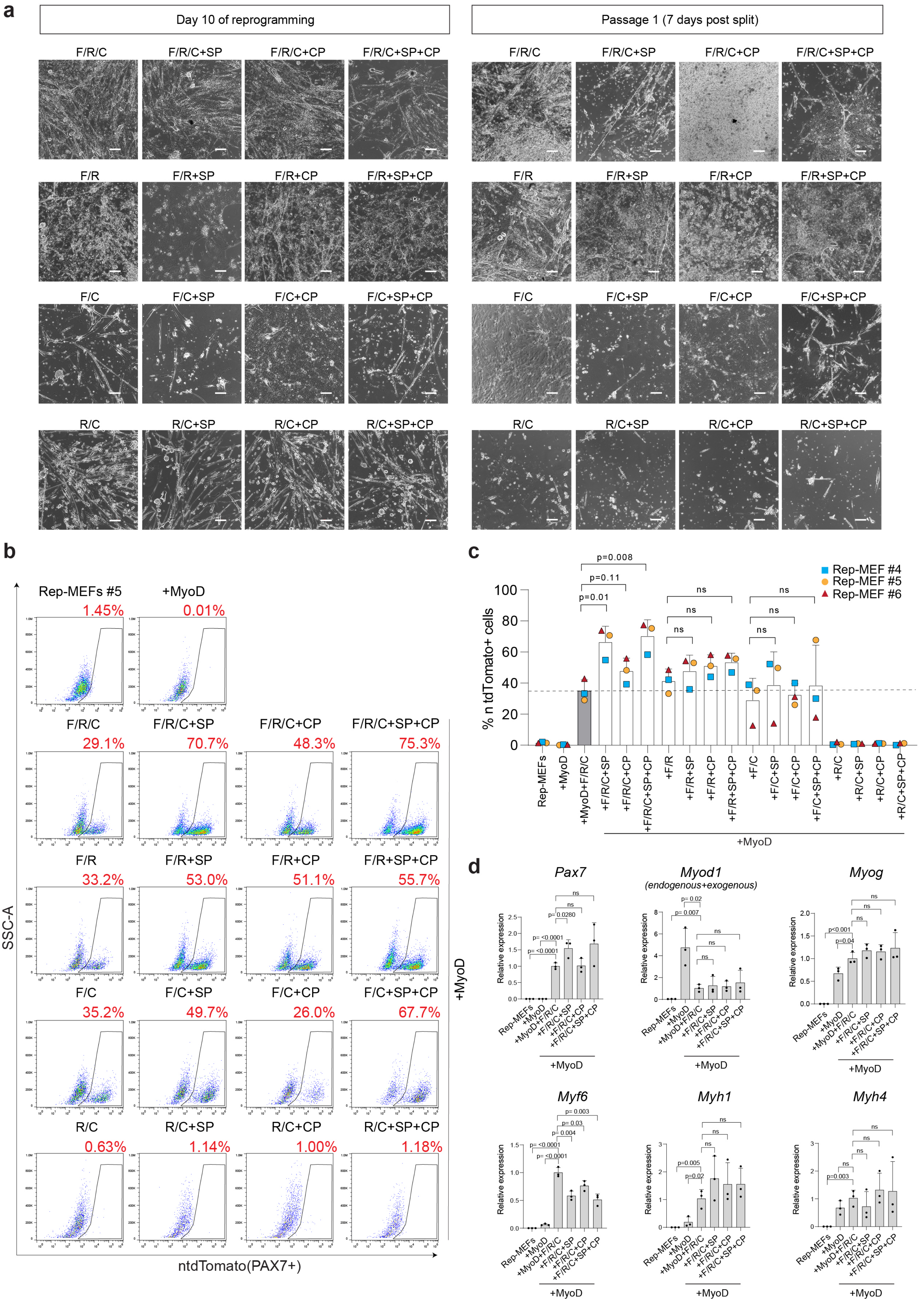

Supplementary Figure 3: Dissecting the effect of small molecule combinations on iMPC derivation

(a) Representative brightfield images of Rep-MEFs exposed to the indicated conditions and times at P1. Scale bar, 200µm. **(b)** FACS plots of *Pax7-CreERT2; R26-LSL-ntdTomato* Rep-MEF #5 showing percentages of PAX7^{+}/ntdTomato^{+} cells at day 10 of reprogramming using the indicated conditions. Cells were labeled with 4-OHT one day prior to analysis. **(c)** FACS-quantification of *Pax7-CreERT2; R26-LSL-ntdTomato* Rep-MEFs on day 10 of reprogramming using the indicated conditions. Cells were labeled with 4-OHT one day prior to analysis. Data are shown as mean±SD. N=3, each dot represents a different cell line. Significance was determined by two-tailed unpaired t-tests. **(d)** RT-qPCR analysis of *Pax7-CreERT2; R26-LSL-ntdTomato* Rep-MEFs #4-6 subjected to the indicated conditions at day 10 of reprogramming. Expression is shown relative to the “+MyoD+F/R/C” condition. Data are shown as mean±SD. N=3, each dot represents a different cell line. Significance is determined by two-tailed unpaired t-tests.

Supplementary Figure 4

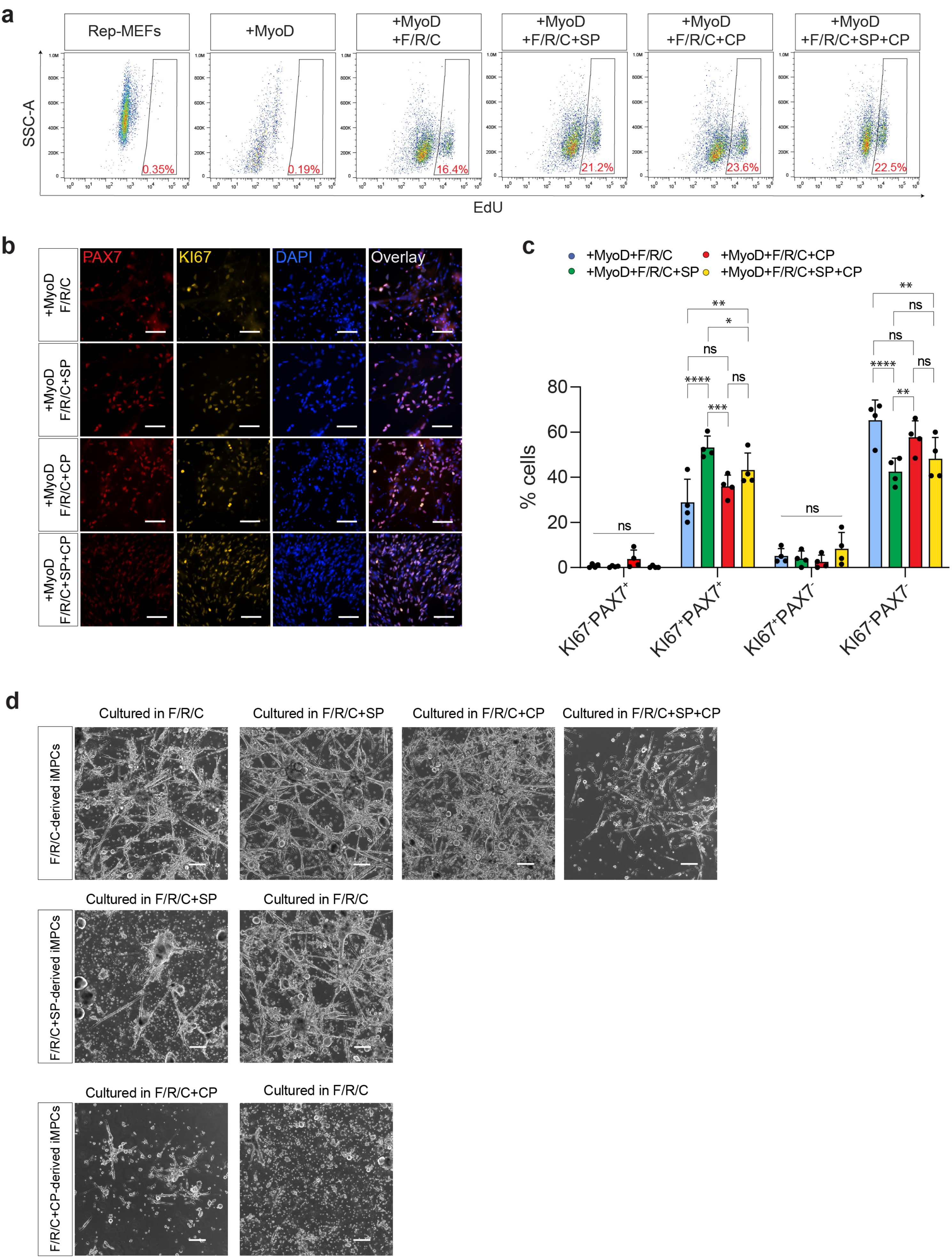

Supplementary Figure 4: Proliferation and growth analysis of iMPCs following compound treatment

(a) FACS plots showing percentages of EdU^+ cells on day 10 of reprogramming of a Rep-MEF clone using the indicated conditions. **(b)** Immunofluorescence images for the indicated conditions in iMPCs at day 10 of reprogramming. Scale bar, 100μm. **(c)** Quantification of (b). Data are shown as mean±SD. N=4, each dot represents a random field of view. Significance was determined by ordinary two-way ANOVA using Tukey's multiple comparisons test. *p<0.05, **p<0.01, ***p<0.001, ****p<0.0001, ns=non-significant. **(d)** Brightfield images of passage 5 iMPCs reprogrammed using the indicated conditions, and maintained for 17 days under the original or an alternative culture condition. Scale bar, 200μm.

Supplementary Figure 5

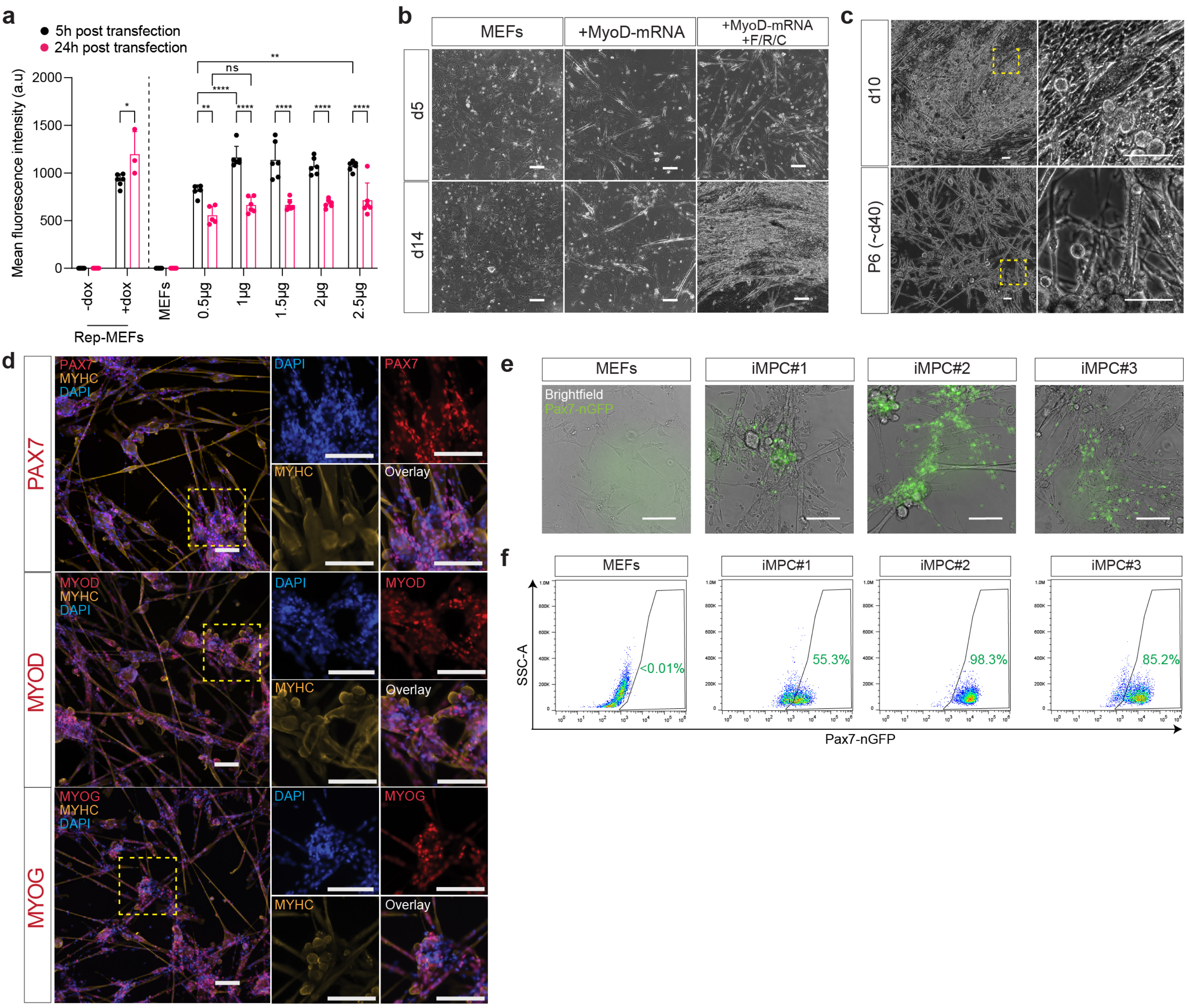

Supplementary Figure 5: Molecular analysis of transgene-free iMPCs

(a) Quantification of mean fluorescence intensity of immunofluorescence images of MyoD-mRNA overexpression. Each data point represents the mean fluorescence intensity of all $MYOD^+$ cells per image. Data are shown as mean±SD. N=3, each dot represents a different cell line. Either 1 or 2 random fields of view were quantified for each cell line. Significance is determined by a mixed-effects analysis using Tukey's multiple comparison's test with a single pooled variance, ns=non-significant; *p<0.05, **p<0.01, ***p<0.001, ****p<0.0001. **(b)** Representative brightfield images of MEFs subjected to the indicated conditions and days. Scale bar, 200μm. **(c)** Brightfield images depicting an emerging transgene-free iMPC clone at day 10 and a stable clone at passage 6. Scale bars, 100μm. **(d)** Representative immunofluorescence images for the indicated markers in a stable transgene-free iMPC clone at passage 5. Scale bars, 100μm. **(e)** Microscopy images showing Pax7-$nGFP^+$ iMPCs between P4-7. Scale bar, 100μm. **(f)** FACS plot analysis of Pax7-$nGFP^+$ transgene-free iMPC clones at P2-3.

Supplementary Figure 6

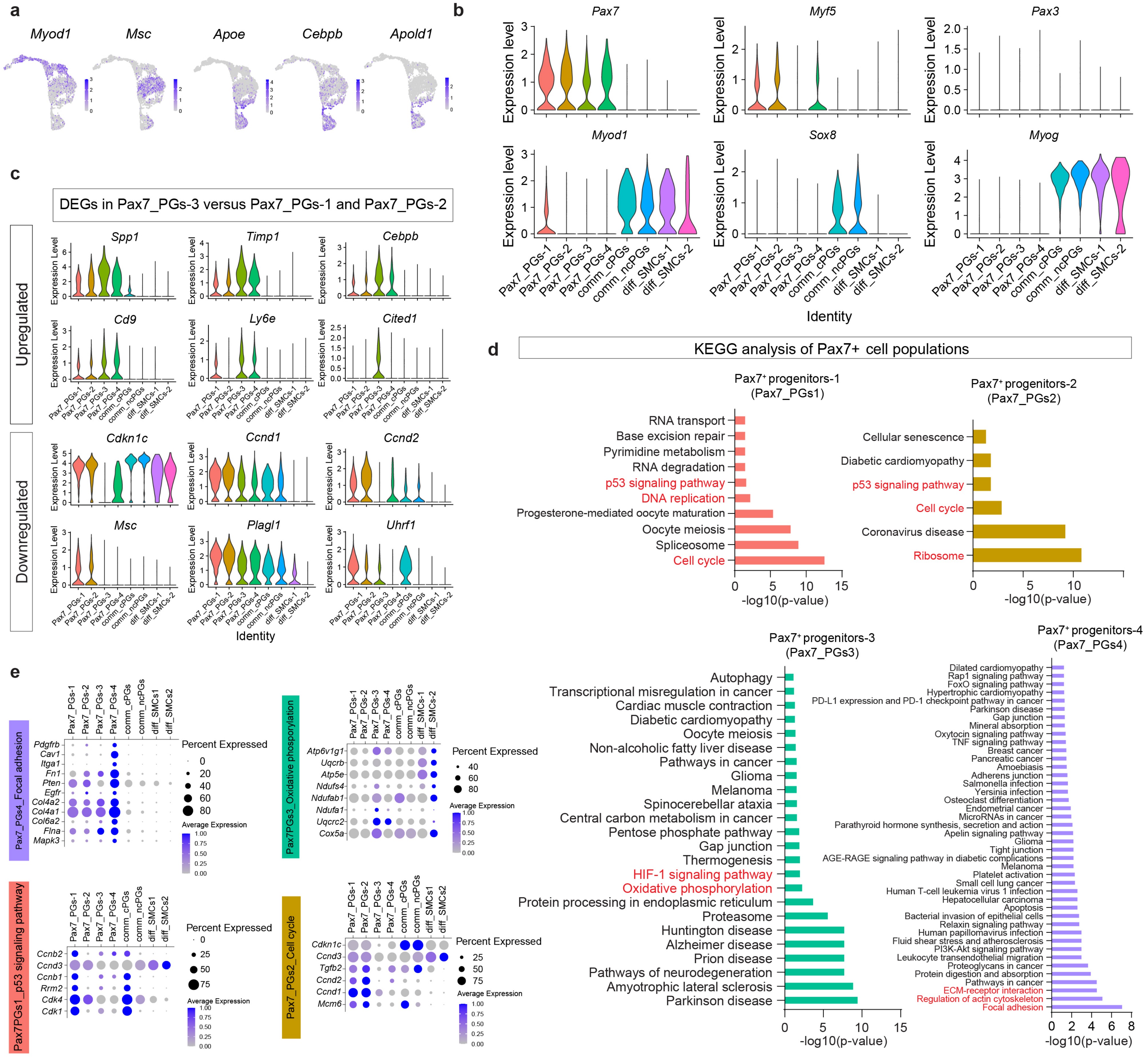

Supplementary Figure 6: scRNA-seq analysis of a transgene-free F/R/C-derived iMPC clone

(a) UMAP projection showing the expression level of selected genes across all cells via a color gradient. **(b)** Violin plots based on scRNA-seq data showing the expression level of key myogenic genes. **(c)** Violin plots based on scRNA-seq data showing the expression level of selected genes that are upregulated (top) and downregulated (bottom) in Pax7_PGs-3 compared to other $Pax7^{+}$ progenitor cell populations. This analysis corresponds to the UMAP shown in Figure 2G. **(d)** A KEGG pathway enrichment analysis based on the marker genes of all the four $Pax7^{+}$ progenitor cell populations (Pax7_PGs 1-4). Only significant pathways are shown with an FDR < 0.05. Pathways of interest are highlighted in red. **(e)** Dot plots demonstrating the expression level of selected genes that are associated with the indicated KEGG pathways for each respective cluster as shown in (d).

Supplementary Figure 7

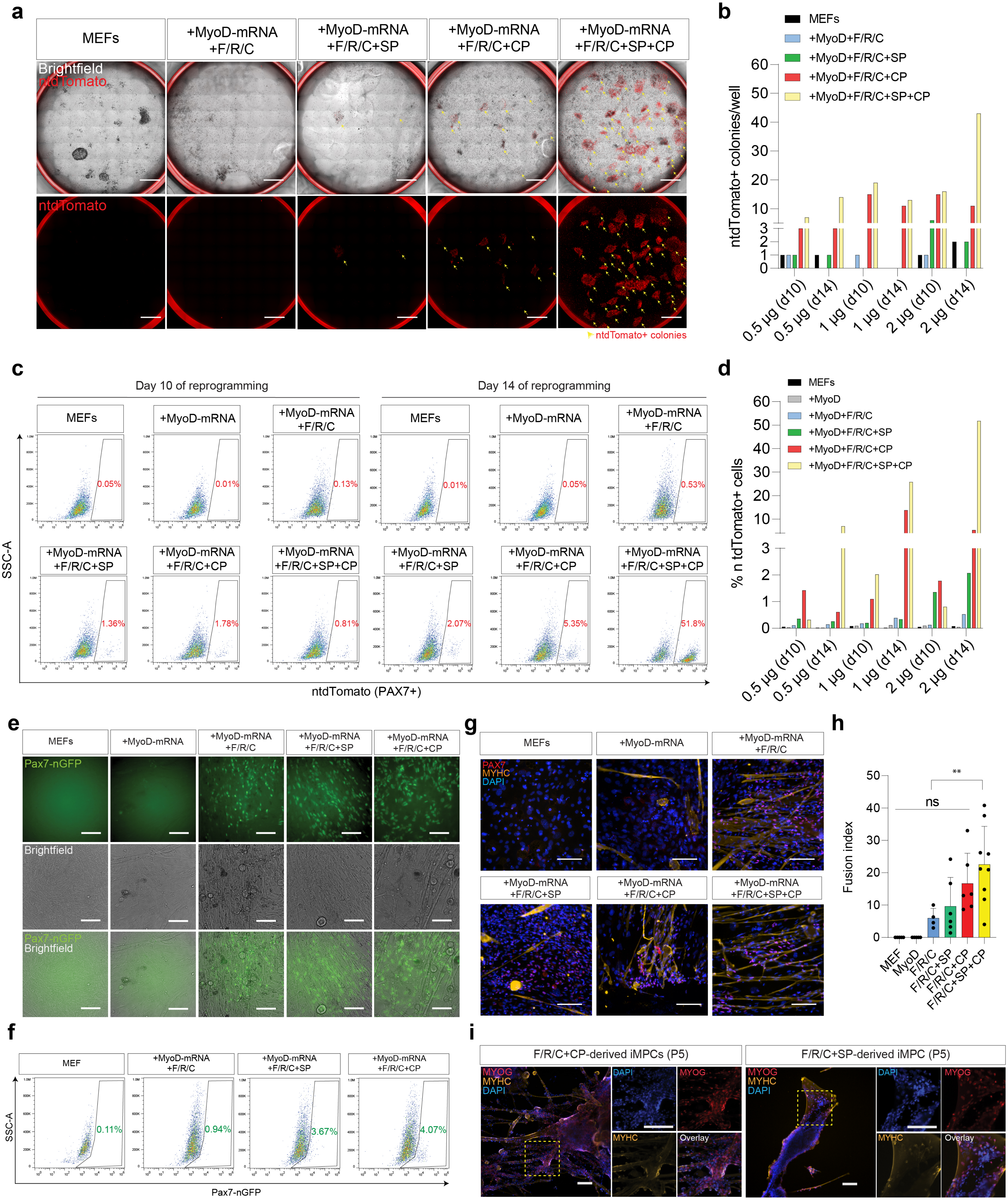

Supplementary Figure 7: Treatment with SP or CP enhance derivation of transgene-free iMPCs

(a) Representative whole-well brightfield and fluorescence images depicting emerging PAX7^{+} colonies 14 days after the start of reprogramming of *Pax7-CreERT2; R26-LSL-ntdTomato* MEFs. Refractory MEFs that do not reprogram using conventional conditions were used in this experiment. Cells were transfected with 2µg MyoD-mRNA during the first 4 days. Emerging iMPC colonies are highlighted with yellow arrowheads. Scale bar, 5mm. **(b)** A graph showing colony quantification during optimization experiment that explored reprogramming with various amounts of MyoD-mRNA in *Pax7-CreERT2; R26-LSL-ntdTomato* MEFs subjected to the indicated conditions and times. N=1 cell line. **(c)** FACS-analysis of *Pax7-Cre-ERT2; R26-LSL-ntdTomato* MEFs subjected to the indicated conditions and times. Shown is an experiment employing transfection of 2µg MyoD-mRNA during the first 4 days. **(d)** A graph showing the percentage of PAX7^{+}/ntdTomato^{+} cells in an experiment that explored reprogramming with various amounts of MyoD-mRNA in *Pax7-Cre-ERT2; R26-LSL-ntdTomato* MEFs subjected to the indicated conditions and times. N=1 cell line. **(e)** Representative brightfield and GFP images of *Pax7-nGFP* MEFs subjected to the indicated reprogramming conditions at day 10 of reprogramming. Scale bar, 100µm. **(f)** FACS plots of *Pax7-nGFP* MEFs subjected to the indicated conditions and analyzed at day 10. **(g)** Representative immunofluorescence images of MEFs exposed to the indicated conditions at day 10 of reprogramming. Scale bar, 100µm. **(h)** Quantification of fusion index during MEF reprogramming via the indicated conditions. Data are shown as mean±SD. N=4-9, each dot represents a random field of view. Significance was determined by an ordinary one-way ANOVA using Dunnett's multiple comparisons test with a single pooled variance taking "MyoD+F/R/C" as control condition, **$p<0.01$, ns=non-significant. **(i)** Representative immunofluorescence images for the indicated markers in stable transgene-free iMPC clones (P5) reprogrammed and kept under either F/R/C+CP or F/R/C+SP conditions. Scale bars, 100µm.

Supplementary Figure 8

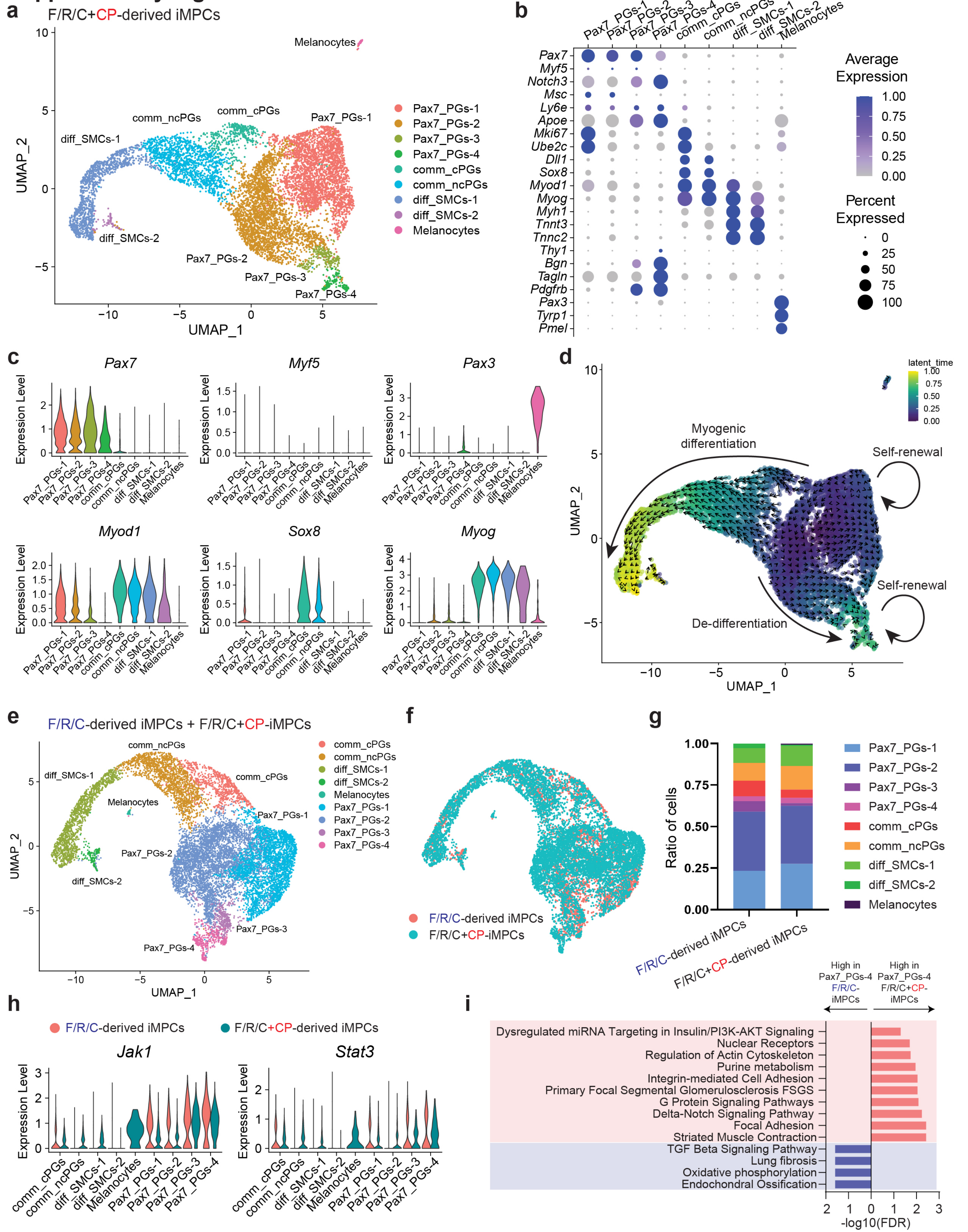

Supplementary Figure 8: scRNA-seq analysis of a stable transgene-free F/R/C+CP-derived iMPC clone

(a) A UMAP projection showing 8,980 cells comprising an iMPC clone at passage 5 that has been generated with MyoD-mRNA and F/R/C+CP treatment. **(b)** Dot plot indicating gene expression level of selected genes in each respective cluster. Average gene expression across all cells in each cluster is shown using a color scale. Percentage of cells expressing the indicated genes is shown as dot size. **(c)** Violin plots showing the expression level of canonical myogenic genes. Note a rare *Pax3*$^{+}$ cell population, most likely denoting melanocytes. **(d)** A UMAP projection of single cell trajectory as indicated by RNA velocity and colored by the latent time of the underlying cellular process. **(e)** Integrated UMAP based on scRNA-seq of +MyoD-mRNA+F/R/C and +MyoD-mRNA+F/R/C+CP-derived stable iMPC clones. **(f)** Integrated UMAPs colored by the indicated iMPC clones. **(g)** Bar plots showing cell population distribution in the indicated iMPC clones. **(h)** Violin plots showing the expression level of representative JAK-STAT pathway-related genes in the indicated samples. **(i)** Pathway enrichment analysis of the Pax7-PGs-4 cell population shown in (e) based on the Wikipathway database. Only significant pathways are shown (FDR < 0.05).

Supplementary Figure 9

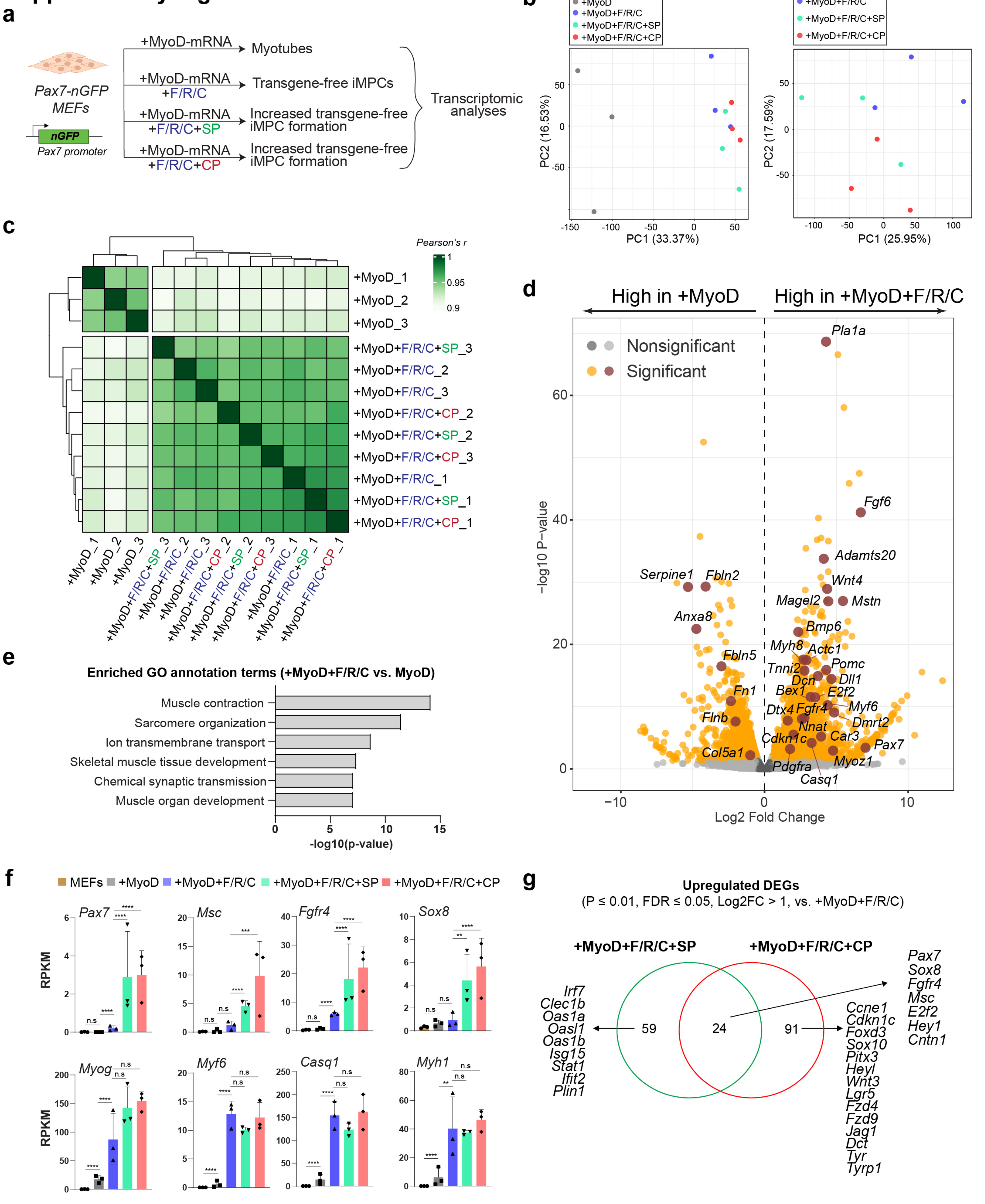

Supplementary Figure 9: Dissecting the effect of SP and CP treatment on iMPC derivation using bulk RNA-seq analysis

(a) A schematic depicting experimental design. **(b)** PCA based on bulk RNA-seq data of the indicated conditions at day 10 of reprogramming. PCs were determined using all genes across samples. N=3, each dot represents a different cell line. **(c)** Normalized gene counts-based correlation matrix across all the indicated replicates and conditions at day 10 of reprogramming. *Pearson's* correlation coeffiecient *r* was used as the color gradient. N=3, three different cell lines were used for this analysis. **(d)** A volcano plot based on bulk RNA-seq data showing DEGs between the indicated conditions at day 10 of reprogramming. Significant DEGs were defined using $|log2FC| > 0.5$ and $FDR < 0.05$. **(e)** Gene ontology analysis of the indicated comparison. Only significant biological processes are shown, p.adjust ≤ 0.01. **(f)** Bar graphs showing RPKM expression level of the indicated genes and reprogramming conditions at day 10 of reprogramming. The data are shown as mean±SD. N=3, each dot represents a different cell line. Statistical significance refers to p-value calculated between the indicated comparisons, **p<0.01, ***p<0.001, ****p<0.0001, ns=non-significant. **(g)** A Venn diagram showing the overlap of upregulated genes based on RNA-seq at day 10 of reprogramming between the indicated comparisons. Only select and significantly upregulated protein-coding genes are shown. Definition of DEGs was based on log2FC > 1, p-value ≤ 0.01 and FDR ≤ 0.05.

Supplementary Figure 10

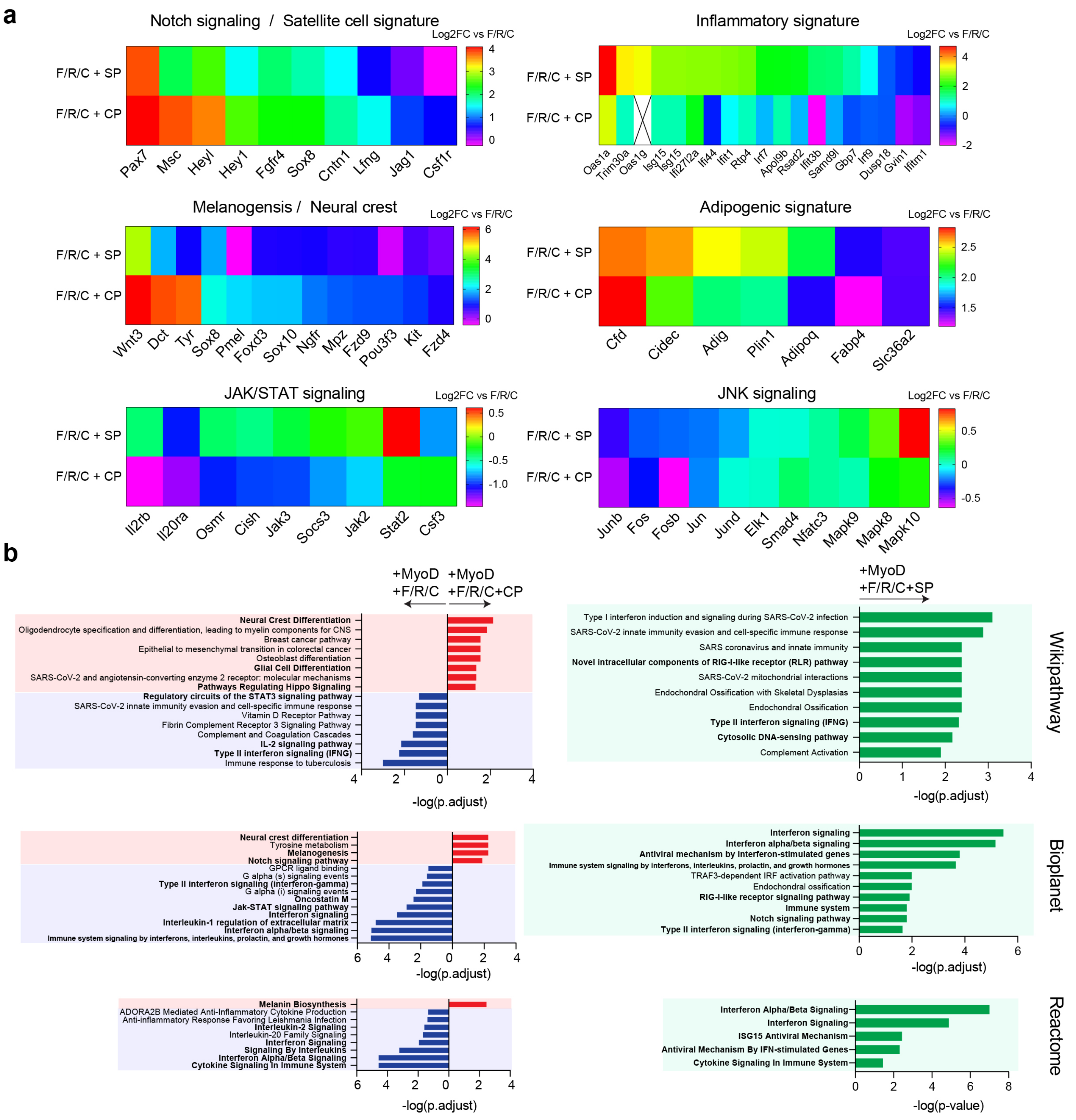

Supplementary Figure 10: Functional annotation analysis based on bulk RNA-seq

(a) Heatmaps showing relative gene expression levels related to relevant biological processes in comparison between F/R/C and the indicated conditions at day 10 of reprogramming. **(b)** ORA using the indicated databases and conditions at day 10 of reprogramming. Only significant pathways are shown (p.adjust < 0.05). Terms of interest are highlighted in bold.

Supplementary Figure 11

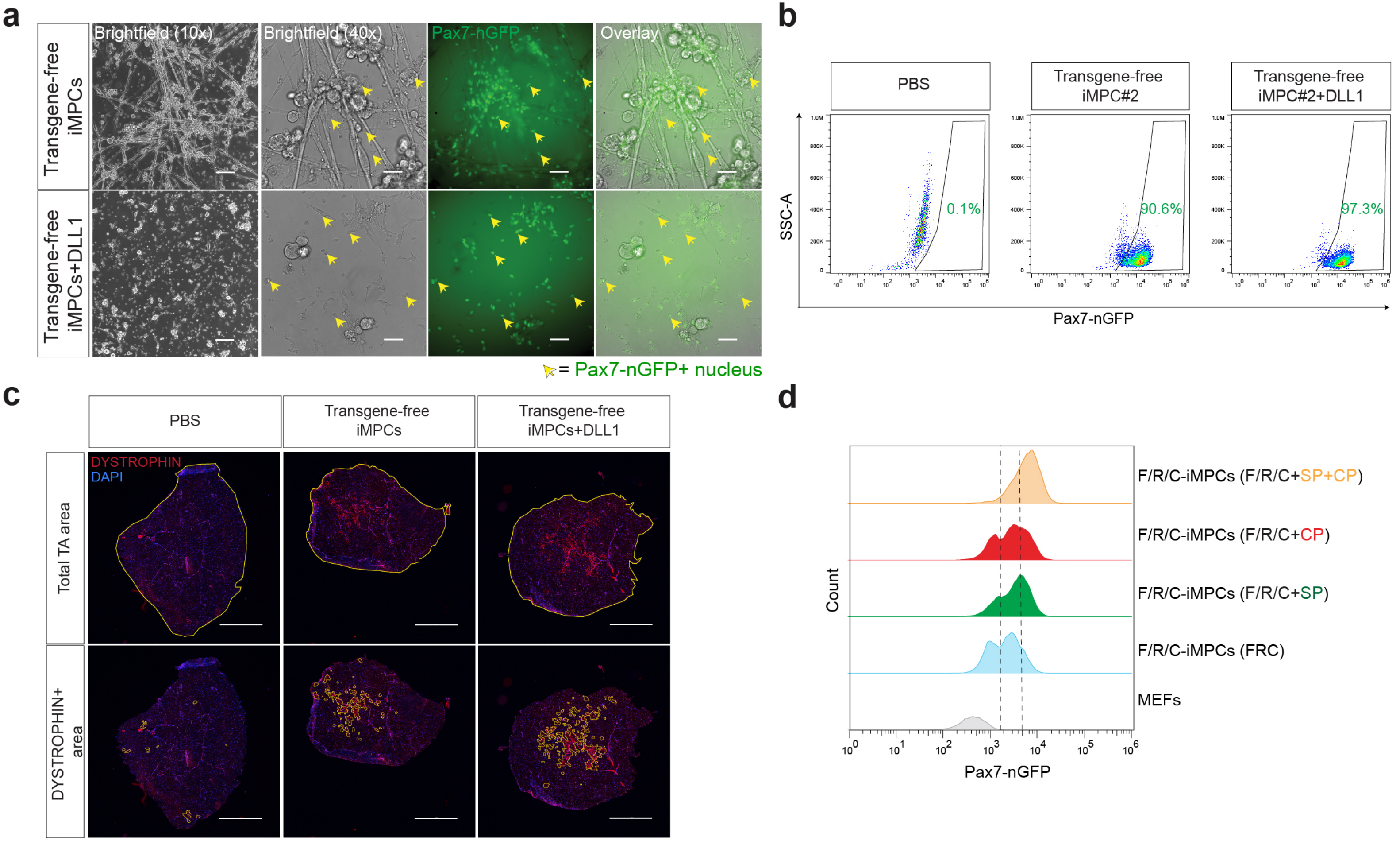

Supplementary Figure 11: Intramuscular transplantation of transgene-free iMPCs

(a) Brightfield and fluorescence images of transgene-free *Pax7-nGFP* iMPCs cultured with and without DLL1 for 5 days. Arrowheads indicate Pax7-nGFP^{+} cells. Scale bar, 200μm (10x) or 50μm (40x). **(b)** Flow cytometric analysis of transgene-free iMPCs cultured on plastic or DLL1-coated plates and analyzed after 5 consecutive days of culture. **(C)** Representative immunofluorescence images of DYSTROPHIN in TA muscles around 4 weeks post cell transplantation. Shown is the quantification method of total DYSTROPHIN^{+} TA muscle area as calculated by the software ImageJ. Scale bar, 1mm. **(d)** FACS histograms showing GFP fluorescence intensity in the indicated conditions at day 10 of reprograming. Related to Figure 4G.

Supplementary Video 1

This movie shows a contractile transgene-free iMPC clone produced by MyoD+F/R/C at passage 3.

Supplementary Tables

Supplementary Table 1 - RT-qPCR probe sequences

The following probes were used in this study:

Gene	Primer sequence
Gapdh	Forward: GTGGAGTCATACTGGAACATGTAG<br>Reverse: AATGGTGAAGGTCGGTGTG<br>Probe: 56-FAM/TGCAAATGG/ZEN/CAGCCCTGGTG/3IABkFQ/
Pax7	Forward: GAAGAAGTCCCAGCACAGC<br>Reverse: GCTACCAGTACAGCCAGTATG<br>Probe: 56-FAM/CCAAAAACG/ZEN/TGAGCCTGTCCACAC/3IABkFQ/
Myod1	Forward: GACACAGCCGCACTCTT<br>Reverse: GCTCTGATGGCATGATGGAT<br>Probe: 56-FAM/ACGACACCG/ZEN/CCTACTACAGTGAGG/3IABkFQ/
Myh1	Forward: GTGAGCTCTGCGTTGATCT<br>Reverse: GCAGCTCCAAGTTCAGTCT<br>Probe: 56-FAM/AGGCCAAAA/ZEN/TCAAAGAGGTGACCGA/3IABkFQ/
Myog	Forward: GACCGAACTCCAGTGCATT<br>Reverse: CTTGCTCAGCTCCCTCAAC<br>Probe: 56- FAM/AGCCCATGG/ZEN/TGCCCAGTGAAT/3IABkFQ/
Myf6	Forward: CCACGTTTGCTCCTCCTTC<br>Reverse: CCCTACAGCTACAAACCCAAG<br>Probe: 56-FAM/AGGCCCCTG/ZEN/GAATGATCCGAAAC/3IABkFQ/
Myh4	Forward: TGCTGGATCTTACGGAACTTG<br>Reverse: GGACTTGGTGGACAAACTACA<br>Probe: 56-FAM/CTGAGGAGG/ZEN/CTGAGGAACAATCCA/3IABkFQ/

Supplementary Table 2 - Media composition

**Medium**	**Composition**
MEF medium	• DMEM (Thermo Fisher, Cat. #10313021)<br>• 1% MEM NEAA, (Thermo Fisher, Cat. #11140050)<br>• 1% Pen/Strep (Thermo Fisher, Cat #15140122)<br>• 0.1% 2-Mercaptoethanol (Thermo Fisher, Cat. #21985023)<br>• 10% (50ml) FBS (Thermo Fisher, Cat. #10270-106)
iMPC medium	• KnockOut-DMEM (Thermo Fisher, Cat. #10829018)<br>• 1% GlutaMAX, (Thermo Fisher, Cat. #35050061)<br>• 1% NEAA (Thermo Fisher, Cat. #11140050)<br>• 1% Pen/Strep, 10’000 U/ml (Thermo Fisher, Cat. #15140122)<br>• 0.1% 2-Mercaptoethanol (Thermo Fisher, Cat. #21985023)<br>• 10% FBS (50ml) (Thermo Fisher, Cat. #10270-106)<br>• 10% KnockOut Serum Replacement (Thermo Fisher, Cat. #10828028)

Medium	Composition
	• 10ng/ml basic FGF (R&D, Cat. #233-FB)
Differentiation medium	• DMEM (Thermo Fisher, Cat. #41966029)<br>• 1% Pen/Strep (Thermo Fisher, Cat #15140122)<br>• 2% Horse serum (Thermo Fisher, Cat. #16050122)

Supplementary Table 3 - Small molecules used in this study

Small molecule	Abbreviation	Biological function	Concentration	Source / cat#
Forskolin	F	Adenylyl cyclase activator	5µM	R&D systems / #1099
RepSox	R	TGF-βRI inhibitor	5µM	R&D systems / #3742
CHIR 99021	C	GSK3β inhibitor/Wnt activator	3µM	R&D systems / #4423
SP600125	SP	JNK inhibitor	10µM	Tocris /#1496
CP690550 citrate	CP	JAK inhibitor	200nM	Tocris /#4556
PP1	-	Src family kinase inhibitor	10µM	Tocris /#1397
A 83-01	-	TGF-βRI inhibitor	10µM	Tocris /#2939; Sigma-Aldrich /#SML0788
(S)-(+)-Dimethindene maleate	Dimethindene	M2 antagonist	2µM	Tocris /#1425
Rapamycin	-	mTOR inhibitor	10nM	Tocris/#1292
Lithium Chloride	LiCl	GSK3β inhibitor/Wnt activator	1mM	Konrad Hochedlinger lab
Y-27632 dihydrochloride	Y-27	ROCK inhibitor	10µM	Tocris/#1254
EPZ 004777		DOT1L inhibitor	5µM	Tocris/#5567
SB 203580	-	p38 inhibitor	10µM	Tocris#1202
Retinoic acid	-	Retinoic acid receptor agonist	2µM	Tocris/#0695
INK128	-	mTORC1/2 inhibitor	200nM	Medchem Express# HY-13328
PD 0325901	-	MEK1/2 inhibitor	1µM	Tocris/#4192

Small molecule	Abbreviation	Biological function	Concentration	Source / cat#
LDN 193189 dihydrochloride	LDN	BMP4 signaling inhibitor	100nM	Tocris/#6053
Dorsomorphin dihydrochloride	Dorsomorphin	AMPK inhibitor / BMP type 1 receptor inhibitor	0.5µM	Tocris/#3093
Pluripotin	-	ERK1/RasGAP inhibitor	1µM	Tocris/#4433
Bone morphogenic protein 4 (human)	BMP4	Growth factor	10ng/ml	Sigma-Aldrich/ #SRP3016
Insulin-like growth factor 1	IGF1	Growth factor	100ng/ml	Sigma-Aldrich / #I8779
Epidermal growth factor (human)	EGF	Growth factor	10ng/ml	R&D systems / #236-EG
Resveratrol	-	Cyclooxygenase inhibitor / SIRT1 activator	10nM	Tocris /#1418
Valproic acid	VPA	HDAC inhibitor	0.5µM	Lucerna-Chem / #HY-10585
5-Azacytidine	5-aza	DNMT1 inhibitor	5µM	Sigma-Aldrich / #A2385
Tranylcypromine	-	Monoamine oxidase inhibitor, demethylase inhibitor	2µM	Konrad Hochedlinger lab
Trichostatin A	-	HDAC inhibitor	20nM	Sigma-Aldrich /#T1952
Sodium butyrate	-	HDAC inhibitor	100µM	Tocris/#3850

Supplementary Table 4 – Antibodies used in this study

Antibody	application (concentration)	source/cat#
Anti-Pax7 (mouse IgG1)	IF (1:100-1:200); WB (1:1000)	R&D systems/Cat. #MAB1675
Anti-MyoD (mouse IgG1)	IF (1:200)	Invitrogen/Cat. #MA5-12902
Anti-Myog (mouse IgG1)	IF (1:500)	SCBT/Cat. #SC-12732 (clone F5D)
Anti-MyHC (mouse IgG2b)	IF (1:1000); WB (1:1000)	R&D systems/Cat. #MAB4470

**Antibody**	**application (concentration)**	**source/cat#**
Anti-Dystrophin (rabbit IgG)	IF (1:200)	Abcam/Cat. #Ab15277
Anti-GFP (mouse IgG2a)	IF (1:50)	Thermo Fisher/Cat. #A1120
Anti-Ki-67 (rabbit IgG)	IF (1:250)	Thermo Fisher/Cat. #MA514520
Goat anti-rabbit IgG AF 488	IF (1:400)	Thermo Fisher/Cat. #11008
Goat anti-mouse IgG2b AF 546	IF (1:500)	Thermo Fisher/Cat. #A21143
Goat anti-mouse IgG1 AF 546	IF (1:400)	Thermo Fisher/Cat. #A21123
Goat anti-mouse IgG1 AF 647	IF (1:500)	Thermo Fisher/Cat. #A21240
Donkey anti-rabbit IgG AF 546	IF (1:400)	Thermo Fisher/Cat. #10040
Donkey anti-rabbit IgG AF 647	IF (1:400)	Thermo Fisher/Cat. #A31573
Horse anti-mouse IgG, HRP-linked	WB (1:1000)	Cell Signaling/Cat. #7076S

Supplementary Table 5 - Plasmids

**Purpose**	**Plasmid name**	**Source**	**Addgene retrieval code**
Lentivirus packaging vector	pLV delta 8.9	Konrad Hochedlinger Lab	-
Lentivirus envelope vector	pLV VSVG	Konrad Hochedlinger Lab	-
Lentivirus transfer vector	pLV[Exp]-Neo-EF1A>Tet3G	VectorBuilder	#184379
Lentivirus transfer vector	pLV[Tet]-Puro-TRE3G>mMyod1[NM_010866. 2]	VectorBuilder	#184380
DNA template for *in vitro* transcription	pT7[mRNA]-5'UTR:mMyod1[NM_010866.2]:Hba-a1_3'UTR	VectorBuilder	#205026
