# Supplementary material for: Transgene-free direct conversion of murine fibroblasts into functional muscle stem cells
Source: NPJ Regen Med. 2023 Aug 8;8:43. doi: 10.1038/s41536-023-00317-z (PMC10409758; doi:10.1038/s41536-023-00317-z)
Supplement: Supplementary file 3 — 20230710_nr-reporting-summary [file 41536_2023_317_MOESM3_ESM.pdf]

nature portfolio

Corresponding author(s): Ori Bar-Nur

Last updated by author(s): Jul 10, 2023

Reporting Summary

Nature Portfolio wishes to improve the reproducibility of the work that we publish. This form provides structure for consistency and transparency in reporting. For further information on Nature Portfolio policies, see our Editorial Policies and the Editorial Policy Checklist.

Statistics

For all statistical analyses, confirm that the following items are present in the figure legend, table legend, main text, or Methods section.

n/a	Confirmed	
☐	☒	The exact sample size (*n*) for each experimental group/condition, given as a discrete number and unit of measurement
☒	☐	A statement on whether measurements were taken from distinct samples or whether the same sample was measured repeatedly
☐	☒	The statistical test(s) used AND whether they are one- or two-sided<br>*Only common tests should be described solely by name; describe more complex techniques in the Methods section.*
☒	☐	A description of all covariates tested
☐	☒	A description of any assumptions or corrections, such as tests of normality and adjustment for multiple comparisons
☒	☐	A full description of the statistical parameters including central tendency (e.g. means) or other basic estimates (e.g. regression coefficient) AND variation (e.g. standard deviation) or associated estimates of uncertainty (e.g. confidence intervals)
☒	☐	For null hypothesis testing, the test statistic (e.g. *F*, *t*, *r*) with confidence intervals, effect sizes, degrees of freedom and *P* value noted<br>*Give P values as exact values whenever suitable.*
☐	☒	For Bayesian analysis, information on the choice of priors and Markov chain Monte Carlo settings
☒	☐	For hierarchical and complex designs, identification of the appropriate level for tests and full reporting of outcomes
☐	☒	Estimates of effect sizes (e.g. Cohen's *d*, Pearson's *r*), indicating how they were calculated

Our web collection on statistics for biologists contains articles on many of the points above.

Software and code

Policy information about availability of computer code

Data collection	No software was used
Data analysis	fastp v0.20.0, Kallisto v0.46.1, CellRanger v7.0.0 and STARSolo v2.7.8a were used for data processing. The R (v4.1.0) packages DESeq2 v1.34.0, SingleCellExperiment v1.18.0, scuttle v1.6.2, Velociraptor v1.6.0, SoupX v1.5, Seurat v.4.2.1, scvelo v0.2.4 were used for data analysis.

For manuscripts utilizing custom algorithms or software that are central to the research but not yet described in published literature, software must be made available to editors and reviewers. We strongly encourage code deposition in a community repository (e.g. GitHub). See the Nature Portfolio guidelines for submitting code & software for further information.

Data

Policy information about availability of data

All manuscripts must include a data availability statement. This statement should provide the following information, where applicable:

- Accession codes, unique identifiers, or web links for publicly available datasets
- A description of any restrictions on data availability
- For clinical datasets or third party data, please ensure that the statement adheres to our policy

The bulk and single cell RNA sequencing data generated as part of this study are available in gene expression omnibus (GEO) with the accession number GSE208064. The weblink to GEO dataset is: https://www.ncbi.nlm.nih.gov/geo/query/acc.cgi?acc=GSE208064 (token for reviewers: epsnieacttwbril)

Human research participants

Policy information about studies involving human research participants and Sex and Gender in Research.

Reporting on sex and gender	*Use the terms sex (biological attribute) and gender (shaped by social and cultural circumstances) carefully in order to avoid confusing both terms. Indicate if findings apply to only one sex or gender; describe whether sex and gender were considered in study design whether sex and/or gender was determined based on self-reporting or assigned and methods used. Provide in the source data disaggregated sex and gender data where this information has been collected, and consent has been obtained for sharing of individual-level data; provide overall numbers in this Reporting Summary. Please state if this information has not been collected. Report sex- and gender-based analyses where performed, justify reasons for lack of sex- and gender-based analysis.*
Population characteristics	*Describe the covariate-relevant population characteristics of the human research participants (e.g. age, genotypic information, past and current diagnosis and treatment categories). If you filled out the behavioural & social sciences study design questions and have nothing to add here, write "See above."*
Recruitment	*Describe how participants were recruited. Outline any potential self-selection bias or other biases that may be present and how these are likely to impact results.*
Ethics oversight	*Identify the organization(s) that approved the study protocol.*

Note that full information on the approval of the study protocol must also be provided in the manuscript.

Field-specific reporting

Please select the one below that is the best fit for your research. If you are not sure, read the appropriate sections before making your selection.

☒ Life sciences ☐ Behavioural & social sciences ☐ Ecological, evolutionary & environmental sciences

For a reference copy of the document with all sections, see nature.com/documents/nr-reporting-summary-flat.pdf

Life sciences study design

All studies must disclose on these points even when the disclosure is negative.

Sample size	The majority of experiments was performed with at least 3 cell lines.
Data exclusions	Data were not excluded from this study. For group-wise comparison in Figure 1e we excluded the "Rep-Mefs" and "+MyoD" conditions conditions from the statistical analysis. This is justified by the one-sided nature of the experiment where we specifically focused on enhancers of reprogramming.
Replication	Findings regarding mRNA and small molecule-mediated reprogramming were replicated repeatedly throughout the duration of the study.
Randomization	For transplantation experiments, we chose male animals as cell recipients to avoid potential Y chromosome-directed graft rejection in female animals. Within the transplant group, animals were chosen randomly to receive either induced myogenic progenitor cells or PBS control injections.
Blinding	Blinding was not relevant to this study.

Reporting for specific materials, systems and methods

We require information from authors about some types of materials, experimental systems and methods used in many studies. Here, indicate whether each material, system or method listed is relevant to your study. If you are not sure if a list item applies to your research, read the appropriate section before selecting a response.

Materials & experimental systems

n/a	Involved in the study
☐	☒ Antibodies
☐	☒ Eukaryotic cell lines
☒	☐ Palaeontology and archaeology
☐	☒ Animals and other organisms
☒	☐ Clinical data
☒	☐ Dual use research of concern

Methods

n/a	Involved in the study
☒	☐ ChIP-seq
☐	☒ Flow cytometry
☒	☐ MRI-based neuroimaging

Antibodies

Antibodies used

anti-Pax7 (mouse IgG1) - IF (1:100-1:200); WB (1:1000)- R&D systems/Cat. #MAB1675
anti-MyoD (mouse IgG1) - IF (1:200) - Inivitrogen/Cat. #MA5-12902
anti-Myog (mouse IgG1) - IF (1:500) - SCBT/Cat. #SC-12732 (clone F5D)
anti-MyHC (mouse IgG2b)- IF (1:1000); WB (1:1000) - R&D systems/Cat. #MAB4470
anti-Dystrophin (rabbit IgG) - IF (1:200) - Abcam/Cat. #Ab15277
anti-GFP (mouse IgG2a) - IF (1:50) - Thermo Fisher/Cat. #A1120
anti-Ki67 (rabbit IgG) - IF (1:250) - Thermo Fisher/Cat. #MA514520
Goat anti-rabbit IgG AF 488 - IF (1:400) - Thermo Fisher/Cat. #11008
Goat anti-mouse IgG2b AF 546 - IF (1:500) - Thermo Fisher/Cat. #A21143
Goat anti-mouse IgG1 AF 546 - IF(1:400) - Thermo Fisher/Cat. #A21123
Goat anti-mouse IgG1 AF 647 - IF (1:500) - Thermo Fisher/Cat. #A21240
Donkey anti-rabbit IgG AF 546 - IF (1:400) - Thermo Fisher/Cat. #10040
Donkey anti-rabbit IgG AF 647 - IF (1:400) - Thermo Fisher/Cat. #A31573
Horse anti-mouse IgG, HRP-linked - WB (1:1000) - Cell Signaling/Cat. #7076S

Validation

anti-Pax7 (mouse IgG1) is a well established antibody to detect Pax7 with 28 citations listed on the manufacturer's website (as of July 12 2022)
anti-MyoD (mouse IgG1) is a well established antibody with 18 citations listed on the manufacturer's website (as of July 12 2022)
anti-Myog (mouse IgG1) is a well established antibody with 373 citations listed on the manufacturer's website (as of July 12 2022)
anti-MyHC (mouse IgG2b) is a well established antibody with 73 citations listed on the manufacturer's website (as of July 12 2022)
anti-Dystrophin (mouse IgG2b) is a well established antibody with 317 citations listed on the manufacturer's website (as of July 12 2022)
anti-GFP (mouse IgG2a) is a well established antibody with 460 citations listed on the manufacturer's website (as of April 2023)
anti-Ki67 (rabbit IgG) is a well established antibody with 742 citations listed on the manufacturer's website (as of April 2023)

Eukaryotic cell lines

Policy information about cell lines and Sex and Gender in Research

Cell line source(s)

In this study we used mouse embryonic fibroblasts isolated from E13.5 pregnant mice as a working cell type. Cell lines were not genotyped for sex.

Authentication

Transgenic cell lines were genotyped to confirm the presence of the transgene.

Mycoplasma contamination

All cell lines used were tested negative for mycoplasma.

Commonly misidentified lines (See ICLAC register)

N/A

Animals and other research organisms

Policy information about studies involving animals; ARRIVE guidelines recommended for reporting animal research, and Sex and Gender in Research

Laboratory animals

The following mus musculus strains were used in this study for isolation of primary cells (MEFs) or transplantation purposes: B6.Cg-Pax7tm1(cre/ERT2)Gaka/J (Jax strain number: 017763), B6.Cg-Gt(ROSA)26Sortm75.1(CAG-tdTomato*)Hze/J (Jax strain number: 025106), B10ScSn.Cg-Prkdcscid Dmdmdx/J (Jax strain number: 018018). Transplanted animals included male animals aged 17-37 weeks.

Wild animals

This study did not involve wild animals.

Reporting on sex

Findings are not sex-dependent. Primary cells were used indiscriminate of sex. Transplantations were performed solely on male animals due to potential Y-chromosome-directed allograft rejection in females.

Field-collected samples	The study did not invovle samples collected from the field.
Ethics oversight	Animals were housed in specific-pathogen-free-like conditions according to the the Swiss Federal Law on Animal Protection and approved by the Cantonal Animal Welfare Committee (license numbers ZH108/2018, ZH177/2018 and ZH002/2022)

Note that full information on the approval of the study protocol must also be provided in the manuscript.

Flow Cytometry

Plots

Confirm that:

☒ The axis labels state the marker and fluorochrome used (e.g. CD4-FITC).

☒ The axis scales are clearly visible. Include numbers along axes only for bottom left plot of group (a 'group' is an analysis of identical markers).

☒ All plots are contour plots with outliers or pseudocolor plots.

☒ A numerical value for number of cells or percentage (with statistics) is provided.

Methodology

Sample preparation	Cells were detached using 0.25% Trypsin, centrifuged at 500xg for 5 mins, washed 2x in PBS and strained through a 37.5µm filter prior to FACS analysis.
Instrument	Sony SH800S Cell Sorter
Software	FlowJo Version 10.6.1
Cell population abundance	This study did not include sorting of cells.
Gating strategy	For identifiying cells that are GFP-positive:<br>1. Gate: FSC-A x SSC-A (Size)<br>2. Gate: FSC-A x FSC-H (Singlets)<br>3. Gate: FSC-A x DAPI-A (Live cells)<br>4. Gate: EGFP-A x SSC-A (GFP-positive cells)<br>For identify cells that are ntdTomato-positive:<br>1. Gate: FSC-A x SSC-A (Size)<br>2. Gate: FSC-A x FSC-H (Singlets)<br>3. Gate: FSC-A x DAPI-A (Live cells)<br>4. Gate: tdTomato-A x SSC-A (ntdTomato-positive cells)

☒ Tick this box to confirm that a figure exemplifying the gating strategy is provided in the Supplementary Information.
